# Ethanol Alters DNMT1/3a/3b Expression Profile, Promotes Persistent DNA Hypomethylation in Human Brain Endothelial Cells and Impairs Late Cortical Angiogenesis

**DOI:** 10.1111/jnc.70375

**Published:** 2026-02-10

**Authors:** Michele Siqueira, Matheus Barros, Paula Lacerda Almeida, Luiza dos Santos Heringer, Henrique Rocha Mendonça, Flávia Carvalho Alcantara Gomes, Joice Stipursky

**Affiliations:** ^1^ Laboratório de Neurobiologia Celular, Instituto de Ciências Biomédicas Universidade Federal do Rio de Janeiro Brazil; ^2^ Laboratório de Biologia das Interações Neurovasculares, Instituto de Ciências Biomédicas Universidade Federal Do Rio de Janeiro Brazil; ^3^ Laboratório de Neurodegeneração e Reparo, Faculdade de Medicina Universidade Federal Do Rio de Janeiro Brazil

**Keywords:** alcohol, angiogenesis, blood brain barrier, endothelial cell, epigenetics

## Abstract

Exposure of the embryonic central nervous system (CNS) to drugs of abuse, such as ethanol, induces severe and persistent damage to neural cells, contributing to the development of fetal alcohol spectrum disorders (FASD). Previously, using a mouse model of FASD, we showed that prenatal alcohol exposure (PAE) directly impairs blood–brain barrier (BBB) development by inducing excessive angiogenesis, altering TJ protein and glucose transporter expression, and modifying the endothelial secretome in the neonatal cerebral cortex. Here, we investigated whether ethanol‐induced effects on endothelial cells involve epigenetic reprogramming, specifically through alterations in DNA methylation profiles. Using human brain microcapillary endothelial cells (HBMECs) treated with ethanol, we observed reduced 5‐methylcytosine (5mC) labeling intensity and DNA methyltransferase (DNMT) activity, accompanied by changes in the levels of DNMT1, DNMT3a, DNMT3b, methyl‐CpG binding protein 2 (MeCP2), and vascular endothelial zinc finger 1 (VEZF1). These effects were associated with altered methylation levels at the promoters of BBB‐related genes, including *GLUT1* and *CLDN5*. Notably, ethanol‐induced hypomethylation persisted over a prolonged period, even after ethanol withdrawal in HBMEC cultures. Treatment with S‐adenosylmethionine (SAM) prevented ethanol‐induced hypomethylation in vitro. In vivo, PAE resulted in increased cortical vascular permeability along with persistent vascularization deficits. Together, our findings suggest that ethanol induces long‐lasting changes in endothelial cells that may compromise cerebral vasculature formation and function, with modulation of DNA methylation representing a potential molecular mechanism underlying these effects.

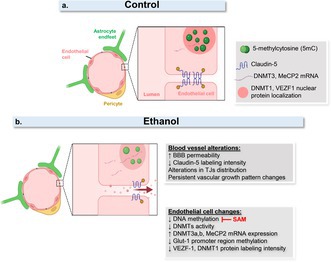

## Graphical abstract

Ethanol exposure alters brain endothelial cell function and blood–brain barrier integrity. In human brain microcapillary endothelial cells (HBMEC) and mouse brain endothelial cells (MBEC) from a prenatal alcohol exposure (PAE) model, ethanol increased DNA methyltransferas‐1 (DNMT1) activity and reduced DNA methylation, affecting DNMT3a/3b, methyl‐CpG binding protein 2 (MeCP2) and vascular endothelial zinc finger 1 (VEZF‐1) expression. Epigenetic changes modified promoter methylation of Glut‐1 and Claudin‐5 genes. PAE increased cortical vascular permeability, altered Claudin‐5 mRNA and disrupted Claudin‐5 protein localization at P0. At P22, vascular development presented reduced cortical vascular arborization. SAM treatment rescued ethanol‐induced DNA methylation changes in HBMEC.

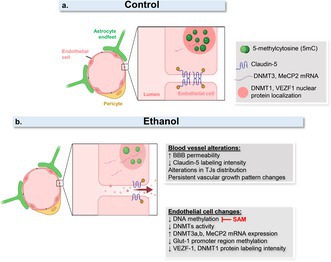

**Abbreviations**
5mC: 5‐methylcytosine
BBB: blood–brain barrier
BDNF: brain‐derived neurothrophic factor
BSA: bovine serum albumin
CNS: central nervous system
DAPI: 4′,6‐diamidino‐2‐phenylindole
DNA: deoxyribonucleic acid
DNMT: DNA methyltransferase
FASD: fetal alcohol spectrum disorder
FBS: fetal bovine serum
FGFb: basic fibroblast growth factor
GAPDH: glyceraldehyde‐3‐phosphate dehydrogenase
GD: gestational day
GLUT‐1: glucose transporter 1
HBMEC: human brain microcapillary endothelial cells
IB4‐FITC: isolectin‐B4 conjugated with fluorescein isothiocyanate
IgG: immunoglobulin
IL‐6: interleukin 6
MBEC: mouse brain endothelial cell
MeCP2: methyl‐CpG binding protein 2
MeDIP: methylated DNA immunoprecipitation
P0: postnatal day 0/newborn
P22: postnatal day 22
PAE: prenatal alcohol exposure
PECAM: platelet endothelial cell adhesion molecule‐1
ROS: reactive oxygen species
RRID: Research, Resource, Identifier
RT‐qPCR: reverse transcription quantitative polymerase chain reaction
TET: ten‐eleven translocation enzyme
TJ: tight junction
TNF‐α: tumor necrosis factor alpha
VE‐cadherin: vascular endothelial cadherin
VEGF: vascular endothelial growth factor
VEGFR2: type 2 vascular endothelial growth factor receptor
VEZF‐1: vascular endothelial zinc finger 1
ZO‐1: *zonnula occludens* 1

## 1 Introduction

Intrauterine exposure to ethanol can induce permanent damage to the developing fetus, resulting in fetal alcohol spectrum disorders (FASD), which include growth restriction and motor, cognitive, and behavioral impairments that persist throughout an individual's life (Jones et al. 1973; Hoyme et al. 2016). Central nervous system (CNS) dysfunctions include reduced volumes of specific brain regions, such as the cerebral cortex, hippocampus, cerebellum, and brainstem, as well as agenesis or hypoplasia of the corpus callosum (Wang et al. 2020; Dodge et al. 2020; Treit et al. 2020). These abnormalities are directly associated with impairments in neural cell survival, differentiation, migration, and trophic factor secretion (Siqueira and Stipursky 2022).

At the cellular level, ethanol affects the survival, differentiation, migration, and protein secretion of glial and neuronal cells (Siqueira and Stipursky 2022). Increasing evidence also suggests that ethanol exposure induces endothelial cell dysfunction and compromises cerebral vascular function (Jégou et al. 2012; Welch et al. 2016; Siqueira and Stipursky 2022).

The blood–brain barrier (BBB) acts as an interface between peripheral blood and the brain and plays a critical role in regulating molecular transport and protecting the neural microenvironment from potentially toxic substances (Abbott et al. 2010; Langen et al. 2019). The BBB is formed by a monolayer of endothelial cells that constitute the capillary wall, closely associated with pericytes, the basement membrane, and astrocytic perivascular endfeet (Obermeier et al. 2013). Endothelial barrier properties are maintained by TJ proteins, including *zonnula occludens*‐1 (ZO‐1) and claudin‐5, which restrict paracellular permeability between the bloodstream and the neural parenchyma. In addition, endothelial cells express multiple transporters that regulate the influx of essential nutrients and the efflux of excess or toxic compounds. Among these, glucose transporter type 1 (GLUT1) is responsible for glucose delivery to the brain (Abbott 2005; Abbott et al. 2010). Loss of GLUT1 expression has been associated with TJ dysregulation, BBB breakdown (Zheng et al. 2010), and impaired angiogenesis (Veys et al. 2020).

Previously, we demonstrated that ethanol treatment of mouse brain endothelial cells increased the global levels of angiogenesis‐related proteins and impaired BBB‐associated functions (Siqueira et al. 2021). More recently, we showed that alcohol consumption during pregnancy alters the proteomic profile of umbilical cord blood serum, leading to deregulation of endothelial barrier properties in brain endothelial cells (Almeida et al. 2025). Together, these findings raise the possibility that ethanol exerts its effects by modulating global gene expression programs in brain endothelial cells.

Epigenetic modifications, particularly DNA methylation, have been identified as key mechanisms underlying neural developmental dysfunctions observed in FASD (Ungerer et al. 2013; Basavarajappa and Subbanna 2016). Alcohol‐induced alterations in DNA methylation have been reported in multiple cell types and brain regions during development (Ungerer et al. 2013). In neural progenitor cells, ethanol exposure increases DNA methyltransferase (DNMT) expression and activity and promotes hypermethylation of promoters of genes involved in cell cycle regulation (Hicks et al. 2010; Miozzo et al. 2018). Prenatal ethanol exposure also disrupts epigenetic maturation by reducing levels of 5‐hydroxymethylcytosine (5hmC), ten‐eleven translocation (TET) enzymes, and methyl‐CpG binding protein 2 (MeCP2), thereby delaying hippocampal granule cell maturation (Chen et al. 2013).

Increasing evidence indicates that DNA methylation is a central regulator of endothelial cell function across tissues, influencing proliferation, differentiation, and angiogenesis (Albig et al. 2007; Goyal and Goyal 2019). During angiogenesis, endothelial gene expression changes are associated with hypomethylation of the promoter region of 515 genes and the hypermethylation of 31 genes (Goyal and Goyal 2019). The role of DNA methylation in angiogenesis is further supported by evidence that hypomethylation in the promoter region of proangiogenic genes, such as the type 2 vascular endothelial growth factor receptor (VEGFR2), promotes cell proliferation and migration and increases angiogenesis (Xu et al. 2017). Moreover, inhibition of MeCP2 in endothelial cells enhances cell survival, angiogenesis, and postischemic blood flow recovery in mice (Rao et al. 2011). In mouse models of cerebral ischemia, DNMT inhibition preserves BBB integrity by increasing expression of endothelial junctional proteins, including ZO‐1 and vascular endothelial cadherin (VE‐cadherin), while reducing pro‐inflammatory cytokines such as tumor necrosis factor‐α (TNF‐α) and interleukin‐6 (IL‐6) in the cerebral cortex (Zeng et al. 2022).

Given accumulating evidence that ethanol triggers teratogenic effects across multiple CNS cell types, we hypothesized that the endothelial dysfunction and angiogenesis deficits previously observed by our group may involve epigenetic mechanisms, specifically alterations in endothelial DNA methylation patterns. Here, we investigated whether human brain endothelial cells and cortical endothelial cells derived from PAE mice exhibit global DNA methylation changes and explored the potential molecular mechanisms underlying these alterations. We demonstrate that ethanol exposure induces DNA hypomethylation in endothelial cells, potentially mediated by reduced DNMT activity and altered levels of epigenetic regulators, including MeCP2 and vascular endothelial zinc finger 1 (VEZF1). In addition, in vitro supplementation with S‐adenosylmethionine (SAM) was effective in preventing the DNA hypomethylation phenotype induced by ethanol. Furthermore, our findings suggest that early‐life ethanol exposure leads to persistent structural abnormalities in the cerebral vasculature, indicating long‐lasting consequences of prenatal ethanol exposure.

## 2 Material and Methods

### 2.1 Animals

Swiss mice were bred and maintained at the animal facility of the Institute of Biomedical Sciences, Federal University of Rio de Janeiro (UFRJ). All experimental procedures were approved by the Institutional Animal Care and Use Committee (CEUA‐CCS) under protocol number 038/24. Pregnant females were housed individually in ventilated cages until delivery or until the litter reached 22 postnatal days. Animals had free access to food and filtered water.

### 2.2 Prenatal Alcohol Exposure (PAE)

Female Swiss mice on gestational day 14 (GD14) received a daily dose of 3.0 g/kg of 30% (v/v) ethanol (Sigma‐Aldrich, Cat. 1009831000) or water (control group) by oral gavage until GD19, as previously described (Siqueira et al. 2021). Control and ethanol‐treated females were allocated based on similar body weight and age. Newborn (P0) and 22‐day‐old (P22) offspring were euthanized by isoflurane overdose (Syntec) using a 5% isoflurane‐soaked cotton placed behind a physical barrier inside a transparent, nonporous container. After cessation of respiration, P0 pups were decapitated, and P22 mice were euthanized by cervical dislocation. Brains were collected and processed for: (1) fixation in 4% paraformaldehyde for 48 h (Vetec, Cat. 30525‐89‐4), sectioning into 40‐μm coronal sections using a vibrating blade microtome (Leica). The sections were processed for immunohistochemistry and analyzed angiogenesis; (2) isolation of the cerebral cortex for RT‐qPCR; and (3) vascular permeability analysis. Animals were obtained from at least three different litters, with 3–6 P0 or P22 mice used per experiment.

### 2.3 Human Brain Endothelial Cell Culture

The human brain microcapillary endothelial cell line (HBMEC) was used with permission from Dr. Dennis Grab (Johns Hopkins University, Department of Pathology, Baltimore, USA). Although HBMEC is not listed as a commonly misidentified cell line by the International Cell Line Authentication Committee, it has not been formally authenticated. This cell line has been used in our previous studies (Siqueira et al. 2018, 2021; Almeida et al. 2025). HBMECs were plated at densities of 1 × 10^4^ cells per well in 24‐well plates or 2 × 10^5^ cells in 25‐cm^2^ flasks and maintained in M199 medium (Thermo Fisher, Cat. 12 340 030) supplemented with 1% glutamine, penicillin–streptomycin (Thermo Fisher, Cat. 10 378 016), and 10% fetal bovine serum (FBS; Thermo Fisher, Cat. 12 657 029). After 5–7 days in vitro, cells were treated with 50 mM or 100 mM ethanol (Sigma‐Aldrich) for 2 or 24 h. These ethanol concentrations correspond to approximately 230 and 460 mg/dL, respectively (Deitrich and Harris 1996), comparable to blood alcohol levels observed in individuals with alcohol dependence (Adachi et al. 1991). Concentrations and exposure times were selected based on previous studies (Haorah et al. 2005; Muneer et al. 2011; Zhang et al. 2014; Siqueira et al. 2021). HBMECs between passages 36 and 38 were used in all experiments (Almeida et al. 2025).

### 2.4 Mouse Brain Endothelial Cell Isolation and Culture

Mouse brain endothelial cells (MBECs) were isolated from the cerebral cortex of P0 Swiss mice as previously described (Siqueira et al. 2018). Briefly, after hypothermia anesthesia, pups were euthanized by decapitation, brains were removed, and cortices were dissected and maintained in ice‐cold DMEM/F12 (Thermo Fisher, Cat. 11330032). Cortical tissue pooled from at least five P0 mice was minced and digested in digestion solution I [DMEM/F12, type II collagenase (1 mg/mL; Gibco, Cat. 17101015), DNase (15 μg/mL; Roche, Cat. 11284932001), and 1% glutamine, penicillin, and streptomycin] for 1 h 30 min at 37°C with agitation. After decantation, undigested material was discarded, and the tissue was resuspended in 20% bovine serum albumin (BSA; Sigma‐Aldrich, Cat. A9418) and centrifuged at 1000 rpm for 20 min. The pellet was subjected to a second digestion with digestion solution II [DMEM/F12, collagenase–dispase (1 mg/mL; Roche, Cat. 12352204), DNase (7 μg/mL)] for 1 h at 37°C. After centrifugation at 1500 rpm for 20 min, the pellet containing microcapillary fragments was resuspended and plated onto Geltrex‐coated 25‐cm^2^ flasks (Thermo Fisher, Cat. A4000046703). Cells were maintained in DMEM/F12 supplemented with 10% FBS, 1.5 ng/mL basic fibroblast growth factor (FGF‐b; Thermo Fisher, Cat. 100‐18B), insulin–transferrin–selenium (Roche, Cat. 11074547001), and 50 μg/mL gentamicin (Thermo Fisher, Cat. 15750060). Cultures were maintained at 37°C in a humidified atmosphere containing 5% CO_2_. Cells were passaged up to passage 3 using 0.125% trypsin (Thermo Fisher, Cat. 25200072) and plated on poly‐L‐lysine–coated surfaces (Sigma‐Aldrich, Cat. P26360). All experiments were performed using cells at passage 2.

### 2.5 Immunocytochemistry

HBMECs grown on coverslips were fixed with 4% paraformaldehyde for 15 min and incubated in blocking solution containing 3% BSA, 5% normal goat serum (NGS; Thermo Fisher, Cat. 31872), and 0.2% Triton X‐100 in PBS for 1 h. Cells were incubated overnight at 4°C with primary antibodies (Table 1), diluted in blocking solution. For DNA methylation analysis, cells were permeabilized with PBS containing 0.5% Triton X‐100 for 5 min and incubated with 4 N HCl for 10 min prior to blocking. Cells were washed and incubated with secondary antibodies (Table 2) for 2 h at room temperature. Nuclei were counterstained with fluorescent nuclear marker 4′,6‐diamidino‐2‐phenylindole (DAPI, Thermo Fisher, RRID:AB_2307445), and coverslips were mounted on glass microscope slides with Fluoromount (Dako). Imaging was performed using a Nikon TE3000 fluorescence microscope or a Leica TCS SP5 confocal microscope.

**TABLE 1:** Primary antibodies.

Antigen	RRID	Host	Dilution	Catalog	Supplier
5‐methylcytosine	AB_2798962	Rabbit	1:300	28692S	Cell Signaling
DNMT1	AB_2537688	Mouse	1:1000	MA5‐16169	Thermo Fisher
Laminin	AB_477163	Rabbit	1:300	L9393	Sigma Aldrich
Claudin‐5	AB_2533200	Mouse	1:500	SC‐365560	Thermo Fisher
VEZF‐1	AB_10846717	Mouse	1:200	SC‐376764	Santa Cruz Biotech.
PECAM (CD‐31)	AB_2801330	Mouse	1:25	SC‐376764	Santa Cruz Biotech.

**TABLE 2:** Secondary antibodies.

Conjugated fluorophore	RRID	Specificity	Dilution	Catalog	Supplier
AlexaFluor 488	AB_143165	Rabbit	1:800	A‐11008	Thermo Fisher
AlexaFluor 546	AB_2534077	Rabbit	1:1000	A‐11010	Thermo Fisher
AlexaFluor 546	AB_2737024	Mouse	1:1000	A‐11030	Thermo Fisher

### 2.6 Immunohistochemistry

Free‐floating brain sections were permeabilized with PBS containing 0.5% Triton X‐100 for 30 min and blocked for 1 h 30 min with agitation. Sections from P22 mice were permeabilized using 1% Triton X‐100 diluted in PBS for 30 min. Sections were incubated overnight at 4°C with Isolectin B4–FITC (Sigma‐Aldrich, Cat. L32895; 1:500) and primary antibodies (Table 1). After washing, sections were incubated with secondary antibodies (Table 2) for 2 h at room temperature, counterstained with DAPI, mounted with Fuoromount on glass microscope slides, and imaged using a Leica TCS SP5 confocal microscope. At least three cortical sections per animal were analyzed.

### 2.7 Reverse Transcription Quantitative Polymerase Chain Reaction (RT‐qPCR)

Cell were lysed with Trizol Reagent (Invitrogen, Cat. 15596026) and total RNA was isolated using Direct‐zol RNA MiniPrep kit (Zymo Research, Cat. R2052). RNA was quantified using the Nanodrop ND‐1000 spectrophotometer (Thermo Fisher) and 1–2 μg of purified RNA was used for cDNA synthesis using RevertAid First Strand cDNA Synthesis Kit (Thermo Fisher, Cat. K1621). cDNA was used for real‐time quantitative PCR (qPCR) reaction using PowerUp SYBR Green Master Mix (Applied Biosystems, Cat. A25780). The reaction cycle was: 95°C for 20 s, followed by 40 cycles of 95°C for 3 s and 60°C for 30 s, 95°C for 15 s, 60°C for 1 min and 95°C for 15 s using QuantStudio 7 Flex Real‐Time PCR System (Applied Biosystems). Relative gene expression levels were calculated using the 2^−ΔΔCT^ method (Livak and Schmittgen, 2001). All reactions were performed in triplicate. The sequences of the oligonucleotides used are described in Tables 3 and 4. The samples were normalized to the endogenous 18S rRNA or glyceraldehyde‐3‐phosphate dehydrogenase (GAPDH) genes.

**TABLE 3:** Oligonucleotides sequences—Mouse.

Claudin‐5 F	CTTCCTGGACCACAACATC
Claudin‐5 R	GCCGGTCAAGGTAACAAA
GAPDH F	AAGAAGGTGGTGAAGCAGGCA
GAPDH R	ACCCTGTTGCTGTAGCCGTATTCA

**TABLE 4:** Oligonucleotides sequences—Human.

DNMT1 F	AGGTGGAGAGTTATGACGAGGC
DNMT1 R	GGTAGAATGCCTGATGGTCTGC
DNMT3a F	CCTCTTCGTTGGAGGAATGTGC
DNMT3a R	GTTTCCGCACATGAGCACCTCA
DNMT3b F	TAACAACGGCAAAGACCGAGG
DNMT3b R	TCCTGCCACAAGACAAACAGCC
MeCP2 F	TGACCGGGGACCCATGTA T
MeCP2 R	CTCCACTTTAGAGCGAAAGGC
18 s F	TTAGAGTGTTCAAAGCAGGCCCGA
18 s R	TCTTGGCAAATGCTTTCGCTCTGG

### 2.8 DNMTs Enzymatic Activity Assay

HBMECs treated with ethanol (50 mM and 100 mM) for 2 h had their cell nuclei extracted using the EpiQuik Nuclear Extraction Kit I (Epigentek, Cat. OP‐0002‐1). The nuclear and cytoplasmic fractions were processed to analyze the protein levels of GAPDH (Abcam, AB_307275) and LaminB1 (Abcam, AB_443298) by western blotting to confirm the purification of the nuclear content respectively. Each sample was loaded in triplicate in acrylamide gel and western blotting was performed as previously described (Siqueira et al. 2018). The purified nuclear fraction was used to analyze DNMT enzymatic activity using the EpiQuik DNA Methyltransferase (DNMT) Activity/Inhibition Assay kit (Epigentek, Cat. P‐3001‐1), according to the manufacturer's recommendation.

### 2.9 Methylated DNA Immunoprecipitation (MeDIP)

HBMEC cells treated with ethanol (50 mM and 100 mM) for 24 h were processed to analyze methylation levels in the promoter regions of specific endothelial genes using the methylated DNA immunoprecipitation (MeDIP) method. After treatment, the cells had their genomic DNA purified using the Wizard Genomic DNA Purification Kit (Promega, Cat. A1120). DNA was measured using the Nanodrop ND‐1000 spectrophotometer (Thermo Fisher) and 1 μg of DNA was fragmented by sonication using 3 pulses of 12 s, followed by 40 s of ice‐cooling between each pulse. The sonicated DNA was heated at 95°C for 2 min to separate the double‐stranded DNA into a single strand to make the methyl groups accessible. Sonicated DNA was processed for enrichment and immunoprecipitation of methylated DNA using a capture antibody to 5‐methylcytosine using the Methylamp Methylated DNA Capture kit (MeDIP; Epigentek, Cat. P‐1015‐48). Sonicated DNA not processed for MeDIP, was used as input DNA. The immunoprecipitated methylated DNA and the input DNA were used for expression analysis by qPCR using primers for the promoter region of endothelial genes (Table 5). MeDIP‐qPCR was calculated by normalizing the Ct values of the immunoprecipitated methylated sample by the Ct values of the input sample (Xu et al. 2017). All reactions were performed in triplicate.

**TABLE 5:** Oligonucleotides sequences for human genes' promoter.

Gene	Sequence
GLUT‐1 F	GGAGAAGTCAATCCCTGGGC
GLUT‐1 R	GACTGGAGTTCTCACTCGGC
Claudin‐5 F	CTCACTGGTAACGAAGCCCC
Claudin‐5 R	AAAGGCAGAAGAGAACCG GG

### 2.10 Chromatin Organization Analysis

Images of 5mC immunostaining were obtained with a Leica TCS SP5 confocal microscope and analyzed by using the NucleusJ 1.0.3 plugin of ImageJ software (RRID:SCR_003070; Poulet et al. 2015). This plugin uses images of individual nuclei in 3D and performs two segmentation steps—nuclear segmentation and chromocenter segmentation. Photomicrographs were acquired using a 63× objective with 2.0 zoom and 1024 × 1024 scan resolution. The segmented images were processed to analyze different parameters related to chromatin organization, such as: number, total volume and fluorescence intensity of chromocenters in relation to the entire nucleus.

### 2.11 Blood Vessels Morphometry In Vivo

Images of coronal sections of the cerebral cortex of newborn (P0) and P22 mice stained with IsolectinB4‐FITC or Laminin were acquired by using a Leica TCS SPE confocal microscope, and blood vessels quantification was performed by using AngioTool software (RRID:SCR_016393, version 0.6a, National Cancer Institute) considering the following parameters: branch points (ratio of vascular branch points/vascular area), vascular density (percentage of area occupied by vessels) and tube length (average length of vessels) (Zudaire et al. 2011).

### 2.12 Vascular Permeability Analysis In Vivo

Brain sections from P0 Swiss mice, either exposed to ethanol via gavage or not, were double‐stained for Laminin and anti‐mouse immunoglobulin (IgG) 488. Images were acquired using a Leica TCS SP5 confocal microscope. Five sequential sections of each animal were used for image acquisition. Additionally, vascular integrity was assessed by using 10‐kDa FITC‐Dextran. Newborn mice were anesthetized with 3% isoflurane vapor and received an intracardiac injection of 4 mg/mL 10‐kDa FITC dextran (Sigma Aldrich, Cat. FD10S), into the left heart ventricle. After 3 min, the animals were euthanized by decapitation, brains were removed, fixed and processed to analyze the colocalization of FITC‐Dextran and vascular markers laminin and claudin‐5 by confocal microscopy (Ben‐Zvi et al. 2014).

### 2.13 Statistics

We used One‐way‐ANOVA test followed by Tukey's posttest, considering a 95% confidence interval. *t* student test was used when comparing 2 groups. *p* Value was considered significant when < 0.05. Data normality was verified by Shapiro–Wilk test. Analysis were carried out using the Graphpad Prism software (version 8.0, RRID:SCR_002798). The data are presented in bar graphs with the treated group means normalized relative to the control group mean, where the controls were considered as 1 or 100. For in vivo experiments, 3–4 pregnant females from the control and ethanol group and 3–4 mouse pups from each litter were used. For in vitro assays, 3–5 independent cultures were used, and experiments were performed in triplicate. Sample size calculations were based in previous publications either for in vivo and in vitro analysis (Siqueira et al. 2018, 2021; Almeida et al. 2025). No test for outliers was conducted. Blinding procedures: One experimenter performed in vivo grouping between experimental groups, ethanol administration and tissue processing, and quantification of different parameters was performed by a different experimenter. For in vitro experiments, no blinding procedures were adopted since each experiment was performed by the same experimenter. The datasets generated during the current study are available from the corresponding author on reasonable request. All statistical data can be assessed in Table S1.

## 3 Results

### 3.1 Prenatal Ethanol Exposure Induces BBB Permeability in Newborn Mice Cerebral Cortex

In previous work, we demonstrated that prenatal alcohol exposure (PAE) induces profound structural alterations in developing cerebral blood vessels, including excessive angiogenesis and changes in the distribution and expression of the TJ protein ZO‐1 and the glucose transporter GLUT1 (Siqueira et al. 2021). To determine whether these structural alterations are associated with functional deficits, we assessed vascular integrity by evaluating permeability to plasma‐derived immunoglobulin G (IgG) and fluorescein isothiocyanate–conjugated dextran (FITC–dextran).

**FIGURE 1:**
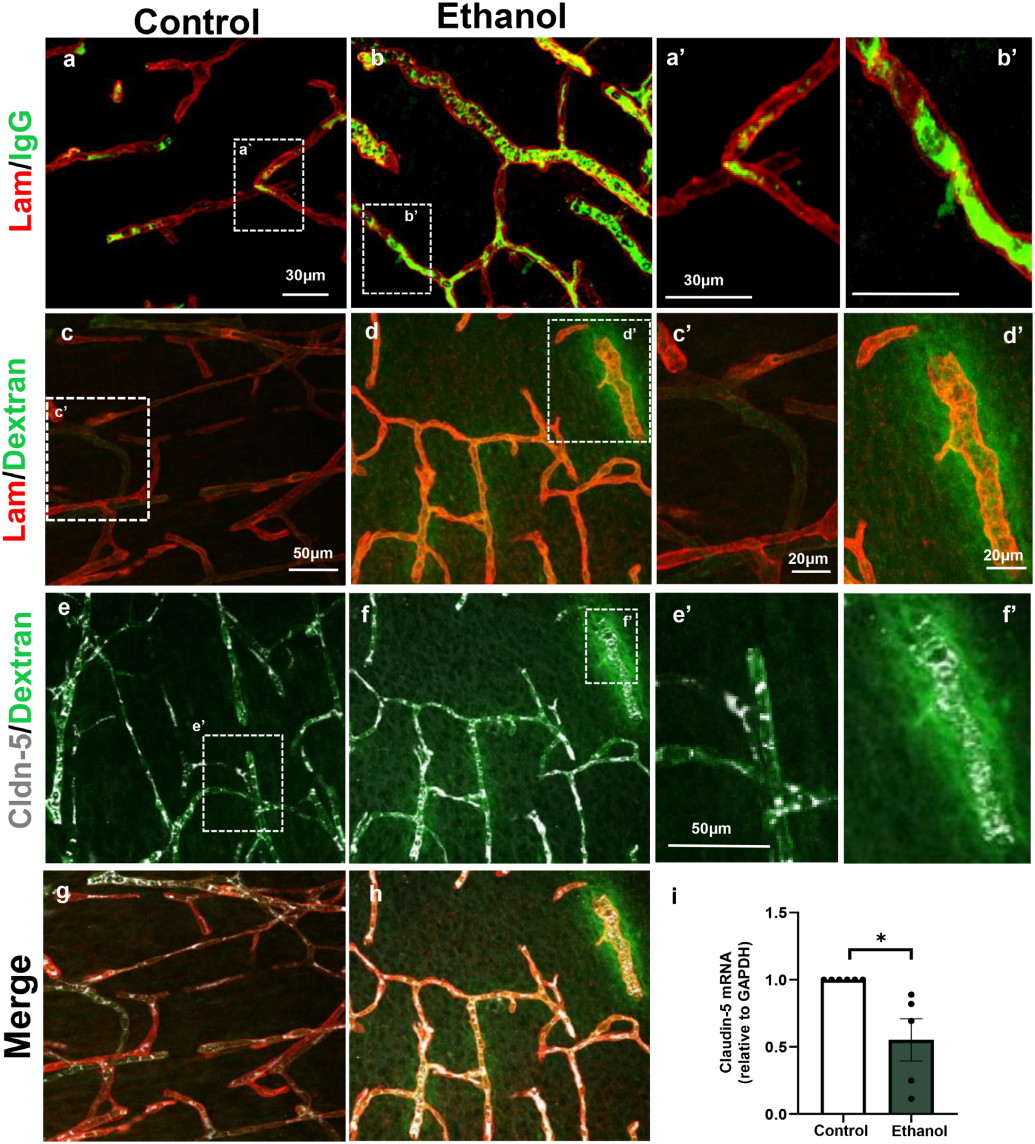
Prenatal ethanol exposure induces BBB permeability. Newborn mice (P0) exposed to ethanol during gestation were subjected to intracardiac injection of 10‐kDa FITC–Dextran. After 3 min of circulation, brains were collected and processed for immunohistochemical analyses. Compared with control animals (a), the cerebral cortex of ethanol‐exposed neonates exhibited extravasation of plasma‐derived IgG (green) into the parenchyma and increased accumulation of IgG within Laminin‐positive blood vessels (red; b, b'). Similarly, ethanol‐exposed animals showed enhanced leakage of FITC–Dextran (green) from laminin‐labeled capillaries (d, d'), whereas in control animals, FITC–Dextran remained confined to the vascular lumen (c, c'). In parallel, a more spread distribution of Claudin‐5 (white) was observed along FITC–Dextran‐labeled vessels in ethanol‐exposed mice (f, f', h), compared with pattern observed in controls (e, e', g). In the cerebral cortex of ethanol‐exposed animals, *Claudin‐5* gene expression was significantly reduced, as determined by RT‐qPCR (i). *p < 0.05. Data are presented as mean ± SEM. *n* = 3–6 pups from 3 independent litters. BBB, blood–brain barrier.

We observed that in ethanol‐exposed animals, Laminin‐labeled cortical blood vessels exhibited focal sites of IgG extravasation, as well as a significant accumulation of IgG signal along the luminal surface, compared with control animals (Figure 1a,a',b,b'). Following intracardiac injection of FITC–dextran, labeling in the cerebral cortex of control mice was restricted to the vasculature (Figure 1c,c'). In contrast, PAE induced the leakage of FITC–dextran from blood vessels, with diffusion into the surrounding brain parenchyma (Figure 1d,d').

Blood vessels increased permeability was corroborated by Claudin‐5 distribution pattern disturbances, which in PAE mice cerebral cortex TJ protein is spread along blood vessels, compared to control condition (Figure 1e–h). Together with Claudin‐5 distribution changes, gene expression was decreased in cerebral cortex of PAE mice, compared to control (Figure 1i).

### 3.2 Ethanol Promotes DNA Hypomethylation in Human Brain Endothelial Cells

Accumulating evidence indicates that multiple neural cell types are direct targets of ethanol‐induced epigenetic reprogramming, resulting in deficits in proliferation and differentiation (Chen et al. 2013; Komada and Nishimura 2022; Bhatia et al. 2023). In addition, dysregulation of angiogenesis and blood vessel maturation has been shown to depend on DNA methylation changes affecting DNA methyltransferases (DNMTs) and the promoter regions of proangiogenic genes (Zhang et al. 2016; Flores et al. 2024).

**FIGURE 2:**
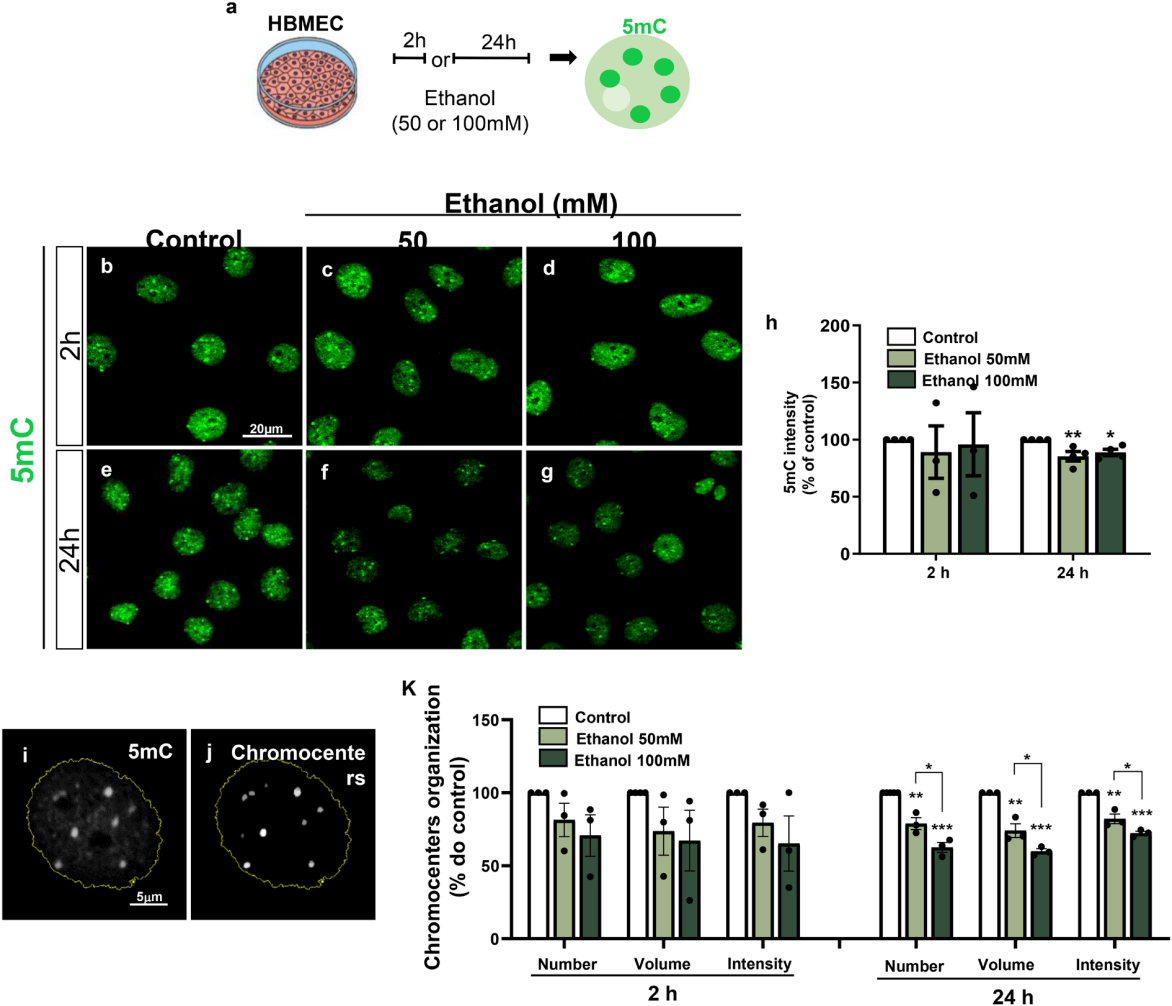
Ethanol promotes DNA hypomethylation in human brain endothelial cells. HBMEC cultures were treated with ethanol (50 or 100 mM) for 2 or 24 h and analyzed by immunocytochemistry for 5‐methylcytosine (5mC; green) (a). Acute exposure to ethanol for 2 h did not significantly alter global 5mC fluorescence intensity (b–d, h) or heterochromatin organization, as assessed by morphometric analyses of chromocenters (i–k). In contrast, 24‐h exposure to ethanol at both concentrations (50 and 100 mM) significantly reduced total 5mC fluorescence intensity (e–h), as well as the number, volume, and fluorescence intensity of chromocenters within the HBMEC nuclei (k). A representative nucleus immunostained for 5mC and processed using the NucleusJ plugin is shown (i). *p < 0.05, **p < 0.01, ***p < 0.001. Data are presented as mean ± SEM. *n* = 3–4 independent cell culture preparations. 5mC, 5‐methylcytosine; HBMEC, human brain microcapillary endothelial cells.

To investigate the impact of ethanol on the DNA methylation profile of endothelial cells, we analyzed global 5‐methylcytosine (5mC) immunolabeling in human brain microcapillary endothelial cells (HBMECs) treated with 50 or 100 mM ethanol for 2 or 24 h (Figure 2a). Acute 2‐h exposure to either ethanol concentration did not significantly alter 5mC fluorescence intensity (Figure 2b–d,h). In contrast, 24‐h treatment with 50 or 100 mM ethanol resulted in significant reductions in 5mC labeling intensity of approximately 15% and 12%, respectively (Figure 2e–g,h).

Given the reduction in global 5mC fluorescence, we performed a more detailed analysis of chromocenter organization by quantifying the number, volume, and labeling intensity of 5mC‐positive chromocenters (Figure 2i,j). Chromocenters are heterochromatic nuclear domains composed of densely packed DNA enriched in epigenetic marks, including DNA methylation at CpG‐rich regions (Probst and Almouzni 2011). Whereas 2‐h ethanol exposure did not affect any of the analyzed chromocenter parameters, 24‐h treatment led to marked reductions in chromocenter number, volume, and fluorescence intensity. Specifically, exposure to 50 mM ethanol reduced these parameters by approximately 20%, while exposure to 100 mM ethanol caused reductions of 38%, 40%, and 28%, respectively (Figure 2k).

### 3.3 Ethanol Alters DNMT1/3a/3b, MeCP2 and VEZF1 Expression

DNA methylation reactions are primarily regulated by DNA methyltransferase (DNMT) enzymes. DNMT1 is responsible for maintaining methylation patterns during DNA replication and cell division, whereas DNMT3a and DNMT3b catalyze *de novo* DNA methylation, establishing new methylation patterns, particularly during cell differentiation and embryonic development (Okano et al. 1999).

**FIGURE 3:**
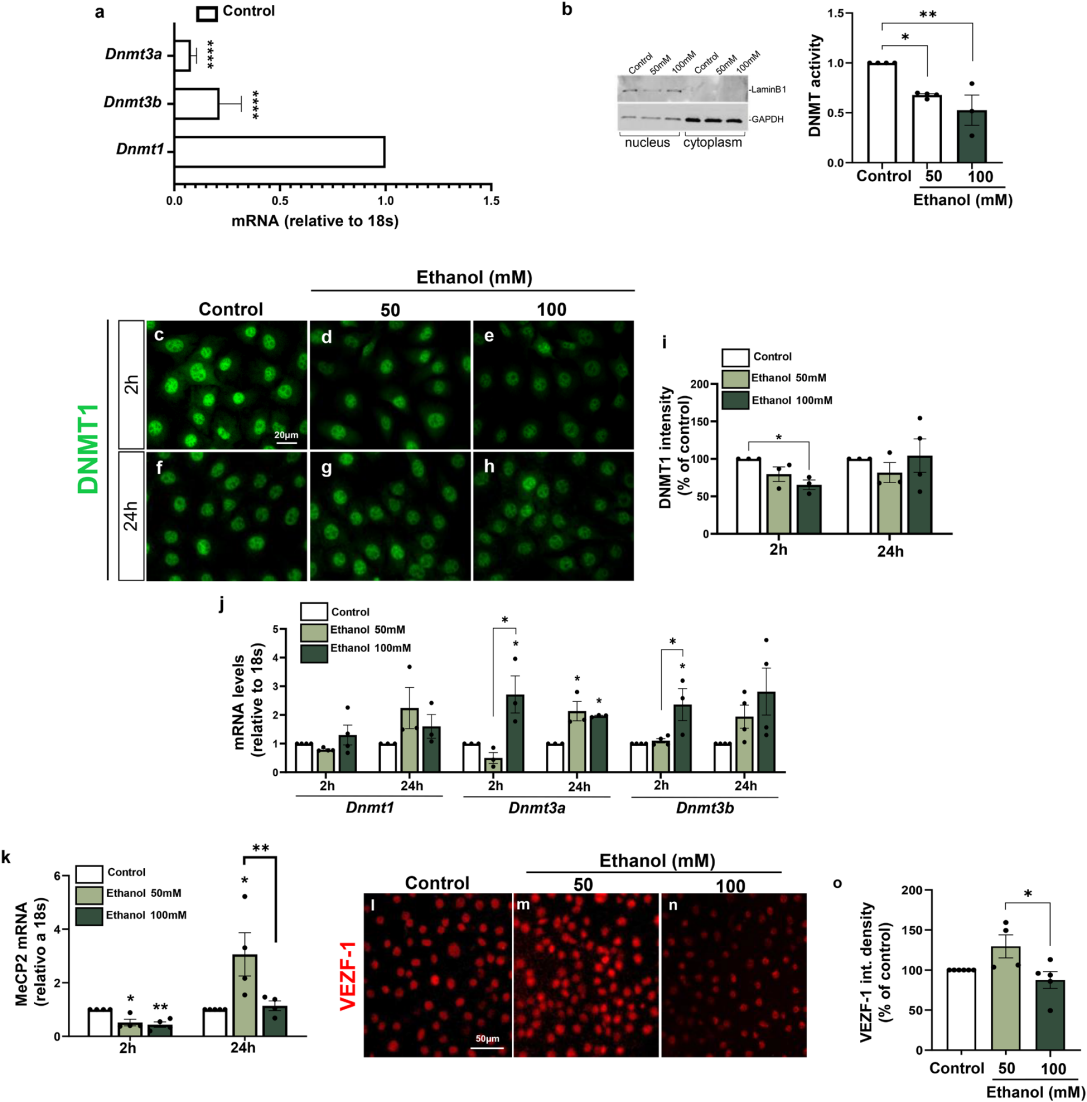
Ethanol alters DNMT1/3a/3b, MeCP2 and VEZF1 expression. HBMEC cultures were treated with different ethanol concentrations (50 or 100 mM) for 2 or 24 h and processed for gene expression analysis by RT‐qPCR, DNMT enzymatic activity assays, and immunocytochemistry. Initially, we found that basal levels of DNMT1 mRNA levels were higher than those of the *DNMT3a* and *DNMT3b* isoforms (a). Nuclear and cytoplasmic protein fractions were isolated, and nuclear protein enrichment was confirmed by immunoblotting for Lamin B1. DNMT enzymatic activity assays performed using nuclear extracts showed that treatment with 50 and 100 mM ethanol significantly reduced DNMT activity (b). Immunocytochemical analyses revealed that 2‐h exposure to 100 mM ethanol decreased DNMT1 fluorescence intensity, an effect that was reversed after 24 h of exposure (c–i). RT‐qPCR analyses showed that *DNMT1* mRNA levels were not significantly altered under any condition; however, exposure to 100 mM ethanol for 2 h increased *DNMT3a* and *DNMT3b* expression, whereas 24‐h exposure selectively increased *DNMT3a* expression without significantly affecting *DNMT3b* (j). Exposure to 50 and 100 mM ethanol for 2 h significantly reduced *MeCP2* mRNA levels, while 24 h after exposure to 50 mM ethanol, we observed an increase in gene expression, and at 100 mM, a recovery to levels similar to those of the control (k). Analysis of VEZF1 immunolabeling showed that 24‐h exposure to 50 mM ethanol increased VEZF1 fluorescence intensity, whereas 100 mM ethanol did not significantly alter this profile, compared to the control (l–o). *p < 0.05, **p < 0.01. Data are presented as mean ± SEM. *n* = 3–5 independent cell culture preparations. DNMT, DNA methyltransferase; HBMEC, human brain microcapillary endothelial cells; MeCP2, methyl‐CpG binding protein 2; VEZF1, vascular endothelial zinc finger 1.

To better understand the mechanisms underlying ethanol‐induced DNA hypomethylation in endothelial cells, we first assessed the basal mRNA expression levels of DNMT isoforms. In control HBMEC cultures, *DNMT3a* and *DNMT3b* accounted for approximately 8% and 20%, respectively, of *DNMT1* expression (Figure 3a). Next, HBMECs were treated with 50 or 100 mM ethanol for 2 h, followed by nuclear and cytoplasmic fractionation, which was validated by Lamin B1 immunoblotting of the nuclear fraction. DNMT enzymatic activity was then measured in nuclear extracts. Treatment with 50 and 100 mM ethanol reduced DNMT activity by approximately 30% and 47%, respectively (Figure 3b).

We next examined the effects of ethanol on DNMT expression. Treatment with 50 mM ethanol did not significantly alter DNMT1 protein fluorescence intensity (Figure 3c–d,f–g,i) or *DNMT1* mRNA levels (Figure 3j). In contrast, exposure to 100 mM ethanol significantly reduced DNMT1 protein fluorescence intensity by approximately 35% after 2 h of treatment, with recovery observed after 24 h (Figure 3c–i), while *DNMT1* mRNA levels remained unchanged (Figure 3j).

We further evaluated the effects of ethanol on *DNMT3a* and *DNMT3b* gene expression. Treatment with 50 mM ethanol increased *DNMT3a* mRNA levels by approximately 2‐fold after 24 h. Moreover, exposure to 100 mM ethanol significantly increased *DNMT3a* and *DNMT3b* mRNA levels by approximately 2.7‐fold and 2.5‐fold, respectively, after 2 h, and increased *DNMT3a* expression by approximately 2.4‐fold after 24 h (Figure 3j).

To further explore ethanol‐mediated modulation of DNA methylation machinery, we analyzed the expression of methyl‐CpG binding protein 2 (MeCP2), a transcriptional regulator that binds methylated CpG sites and recruits corepressor complexes (Zachariah and Rastegar 2012; Vuu et al. 2023). Acute exposure to both ethanol concentrations for 2 h reduced *MeCP2* gene expression by approximately 60%. In contrast, 24‐h exposure to 50 mM ethanol significantly increased *MeCP2* expression by approximately threefold compared with controls, whereas 100 mM ethanol did not induce significant changes (Figure 3k).

Finally, we assessed the effects of ethanol on vascular endothelial zinc finger 1 (VEZF1) expression. VEZF1 is a Krüppel‐like zinc finger protein involved in angiogenesis and vascular development, functioning as a promoter–insulator regulatory factor (Gowher et al. 2008; AlAbdi et al. 2018). Cells treated with 50 mM ethanol for 24 h exhibited a 30% increase in VEZF1 protein fluorescence intensity, whereas treatment with 100 mM ethanol did not significantly alter VEZF1 intensity compared with controls (Figure 3l–o).

### 3.4 Ethanol Alters Methylation Levels of Endothelial Genes'promoter Essential to BBB‐Related Functions

We have shown that ethanol exposure induces a global reduction in 5‐methylcytosine (5mC) levels in endothelial cells and modulates the expression of key epigenetic regulators, including DNMTs, MeCP2, and VEZF1. Based on these findings, we investigated whether ethanol‐induced DNA hypomethylation specifically affects promoter regions of genes involved in angiogenesis and blood–brain barrier (BBB) function.

**FIGURE 4:**
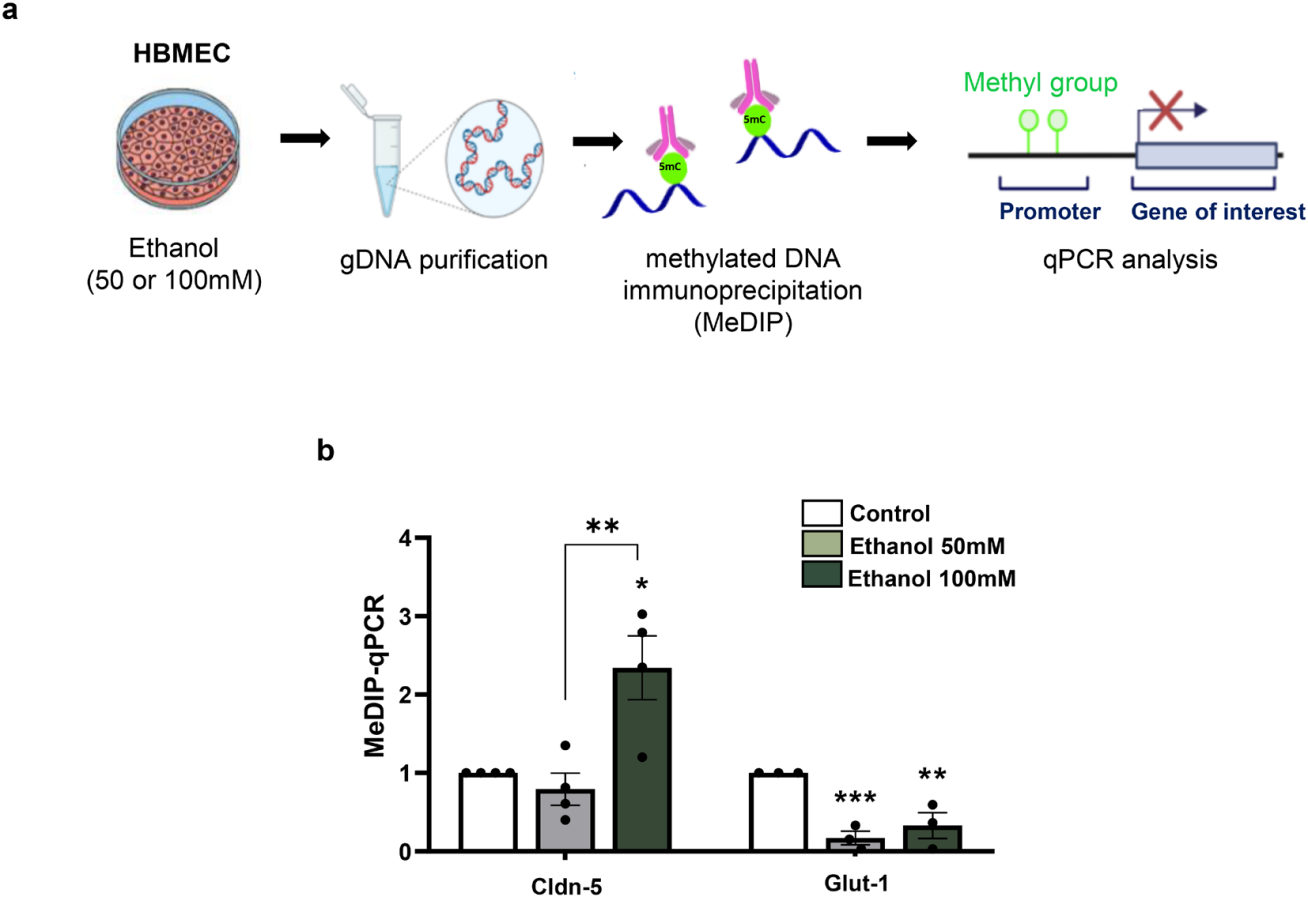
Ethanol alters methylation levels of endothelial genes' promoter essential to BBB‐related functions. HBMEC cultures were treated with ethanol (50 or 100 mM) for 24 h, after which genomic DNA was isolated and methylated regions were immunoprecipitated by antibody binding (MeDIP). The immunoprecipitated DNA was subsequently analyzed by qPCR to assess methylation levels at specific gene promoters (a). Exposure to 50 mM ethanol did not significantly alter methylation levels at the *Claudin‐5* promoter but significantly reduced methylation at the *Glut1* promoter. Treatment with 100 mM ethanol significantly increased methylation at the *Claudin‐5* promoter while reducing methylation at the *Glut1* promoter (b). *p < 0.05, **p < 0.01, ***p < 0.001 Data are presented as mean ± SEM. *n* = 3–4 independent cell culture preparations. HBMEC, human brain microcapillary endothelial cells; Glut1, glucose transporter 1.

Genomic DNA was isolated from HBMEC cultures treated with 50 or 100 mM ethanol for 24 h. Methylated DNA was enriched by methylated DNA immunoprecipitation (MeDIP), followed by quantitative PCR analysis of promoter regions of *Claudin‐5* and *GLUT1* genes (MeDIP–qPCR) (Figure 4a). Methylation levels at the *Claudin‐5* promoter were not significantly altered by exposure to 50 mM ethanol; however, treatment with 100 mM ethanol resulted in an approximately 1.3‐fold increase in promoter methylation. In contrast, exposure to 50 and 100 mM ethanol markedly reduced methylation levels at the *GLUT1* promoter by approximately 5.9‐fold and 3.0‐fold, respectively (Figure 4b).

### 3.5 Ethanol Promotes Persistent Hypomethylation in Endothelial Cells

**FIGURE 5:**
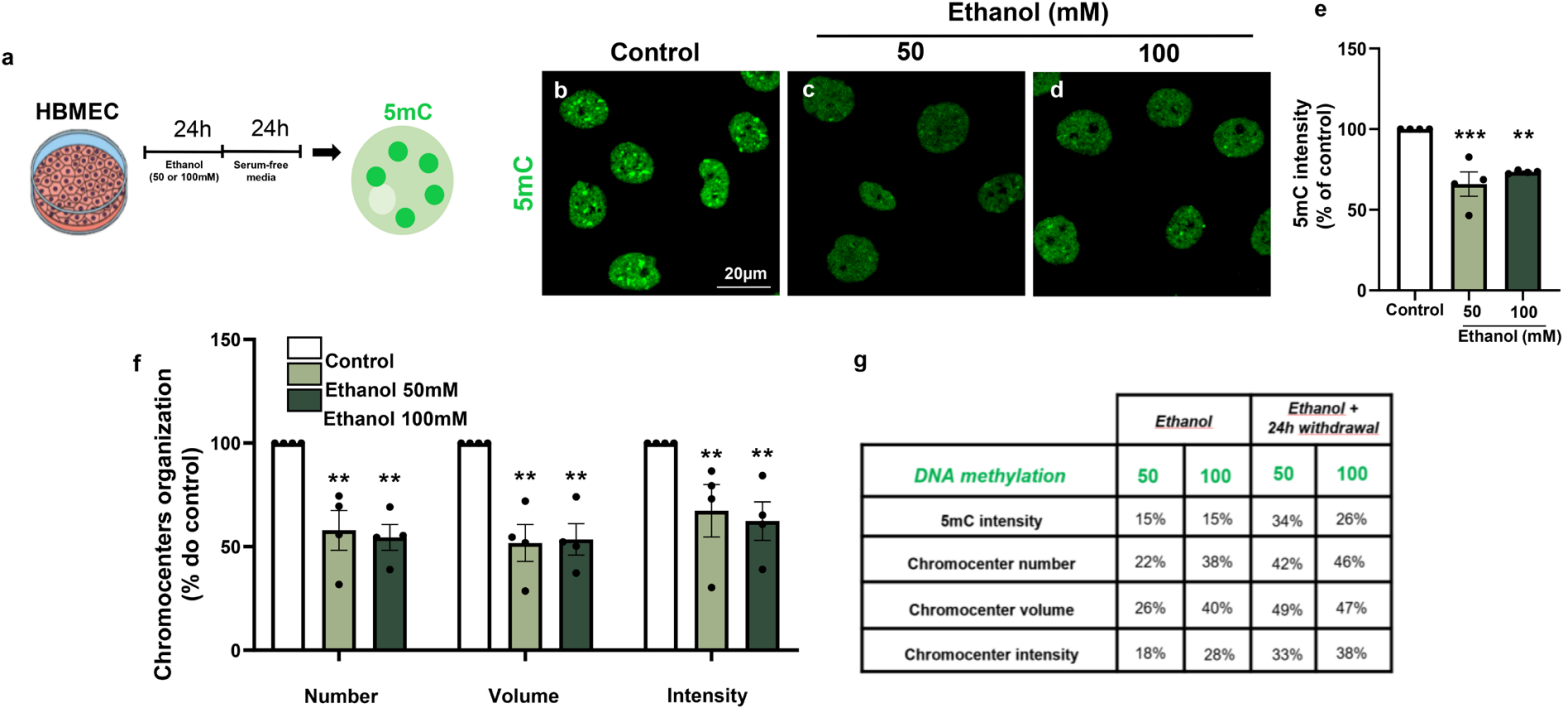
Ethanol promotes persistent hypomethylation in endothelial cells. HBMEC cultures were treated with ethanol (50 or 100 mM) for 24 h, washed, and maintained for an additional 24 h in serum‐free culture medium (a). Immunofluorescence analyses revealed sustained reductions in the fluorescence intensity of 5‐methylcytosine (5mC; green) (b–e), along with decreases in the number, volume, and fluorescence intensity of chromocenters (f). Notably, reductions in global 5mC fluorescence intensity and chromocenter‐associated parameters were more pronounced after the 24‐h withdrawal period than those observed immediately after the initial 24‐h ethanol exposure (g; compare with Figure 2). *p < 0.05, **p < 0.01, ***p < 0.001. Data are presented as mean ± SEM. *n* = 4 independent cell culture preparations. HBMEC, human brain microcapillary endothelial cells; 5mC, 5‐methylcytosine.

To determine whether ethanol‐induced DNA hypomethylation is reversible after ethanol removal or persists over time, HBMEC cultures were treated with ethanol (50 or 100 mM) for 24 h, thoroughly washed, and maintained in fresh culture medium for an additional 24 h (Figure 5a). We observed that endothelial DNA hypomethylation persisted even after ethanol withdrawal, as indicated by sustained reductions in global 5mC labeling intensity of approximately 34% and 26% following exposure to 50 and 100 mM ethanol, respectively (Figure 5b–e).

Persistent alterations in chromatin organization were also observed after ethanol withdrawal. Specifically, the number of chromocenters was reduced by approximately 42% and 46%, chromocenter volume by 49% and 47%, and chromocenter fluorescence intensity by 33% and 38% after exposure to 50 mM and 100 mM followed by 24 h withdrawal, respectively (Figure 5f). Notably, when comparing global 5mC fluorescence intensity and chromocenter organization across treatment paradigms, the magnitude of hypomethylation and chromatin disorganization in cells subjected to ethanol exposure followed by a 24‐h withdrawal period was greater than that observed in cells analyzed immediately after acute ethanol treatment (Figure 5g).

### 3.6 Prenatal Ethanol Exposure Promotes Hypomethylation in Cerebral Cortex Isolated Mouse Brain Endothelial Cells

**FIGURE 6:**
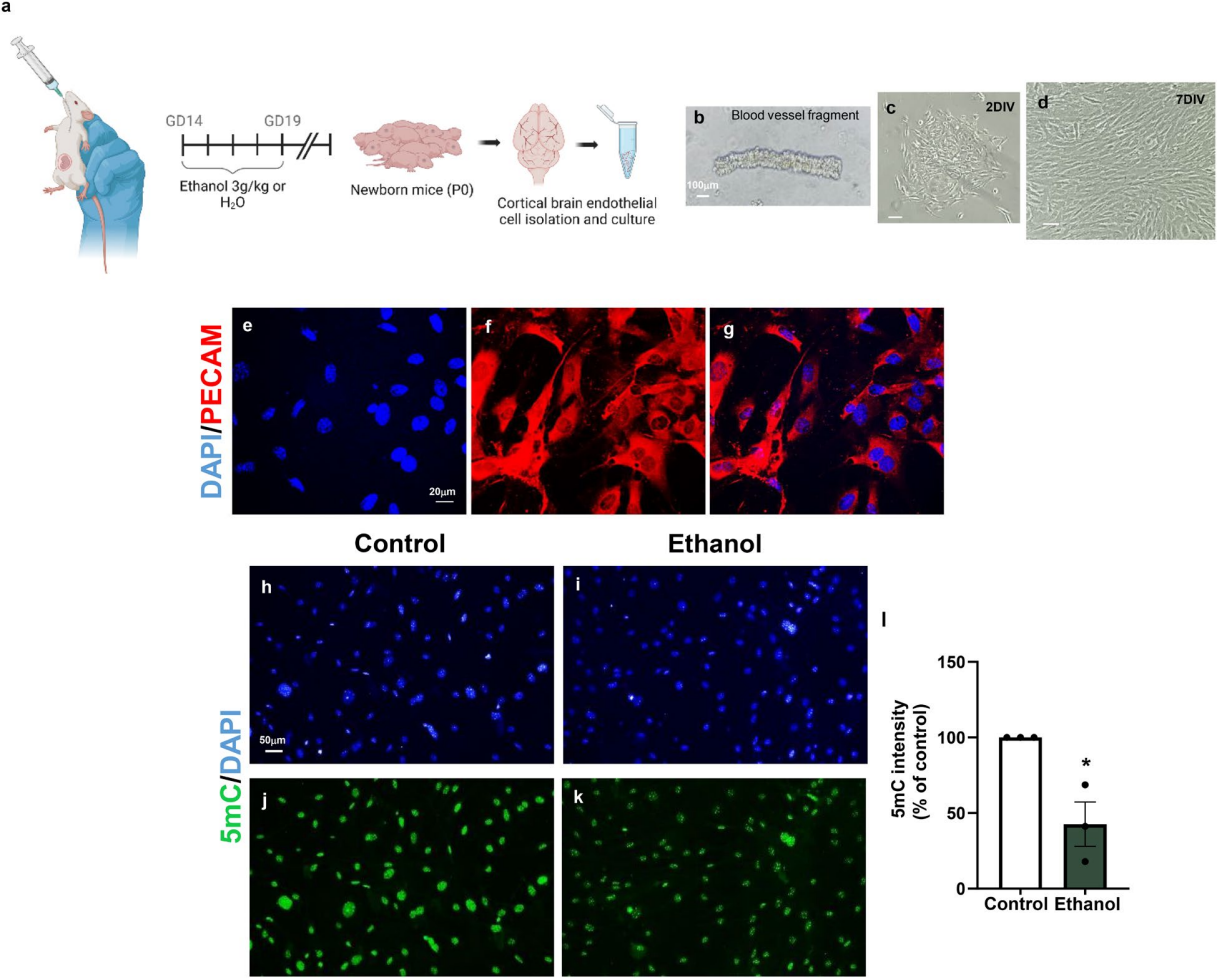
PAE induces hypomethylation in cortical MBECs. Pregnant Swiss mice were exposed to ethanol (3 g/kg) or water (control group) from gestational day 14 (GD14) to GD19. Brains from the offspring were dissected, and the cerebral cortex was processed for the isolation of microvascular fragments (a), which were subsequently plated and cultured until confluence (b–d). The isolated cortical mouse brain endothelial cells (MBECs) were positive for platelet endothelial cell adhesion molecule‐1 (PECAM‐1) (e–g). Immunofluorescence analyses revealed a significant reduction in 5‐methylcytosine (5mC) labeling intensity in MBECs derived from PAE mice compared with controls (h–l). *p < 0.03. Data are presented as mean ± SEM. *n* = 3 independent cell culture preparations from 3 different litters. Each culture was generated from a pool of at least 5 mice. 5mC, 5‐methylcytosine; MBEC, mouse brain endothelial cells; GD, gestational day; PECAM, platelet endothelial cell adhesion molecule‐1.

We previously demonstrated that PAE impairs neurovascular development (Siqueira et al. 2021), and here we show that this event is accompanied by a loss of BBB integrity in the cerebral cortex of newborn mice. To further determine whether these neurovascular dysfunctions are associated with DNA methylation alterations in endothelial cells, we isolated mouse brain endothelial cells (MBECs) from the cerebral cortex of control and PAE‐exposed animals (Figure 6a) and analyzed global 5‐methylcytosine (5mC) fluorescence intensity.

We first confirmed the successful isolation of blood vessel fragments from both control and ethanol‐exposed mice (Figure 6b). MBECs initially formed small colonies within the first 48 h in vitro (Figure 6c) and reached confluence after 7 days in culture (Figure 6d). Immunolabeling for platelet endothelial cell adhesion molecule‐1 (PECAM‐1) confirmed the endothelial identity of the isolated cells (Figure 6e–g).

Analysis of DNA methylation revealed a marked reduction in global 5mC labeling intensity in MBECs derived from PAE mice. Specifically, we observed an approximately 53% decrease in 5mC fluorescence compared with control cells (Figure 6h–l).

### 3.7 Blood Vessels Impairments Induced by PAE Is Observed Later in Development

During the first weeks of postnatal development, the CNS vasculature undergoes extensive remodeling, characterized by rapid vessel growth and branching, followed by refinement through the pruning of unperfused and nonstabilized vessels (Harb et al. 2013; Wälchli et al. 2023). Given that our results demonstrate that PAE induces structural and functional vascular deficits in newborn mice, we next investigated whether these abnormalities persist later in development.

**FIGURE 7:**
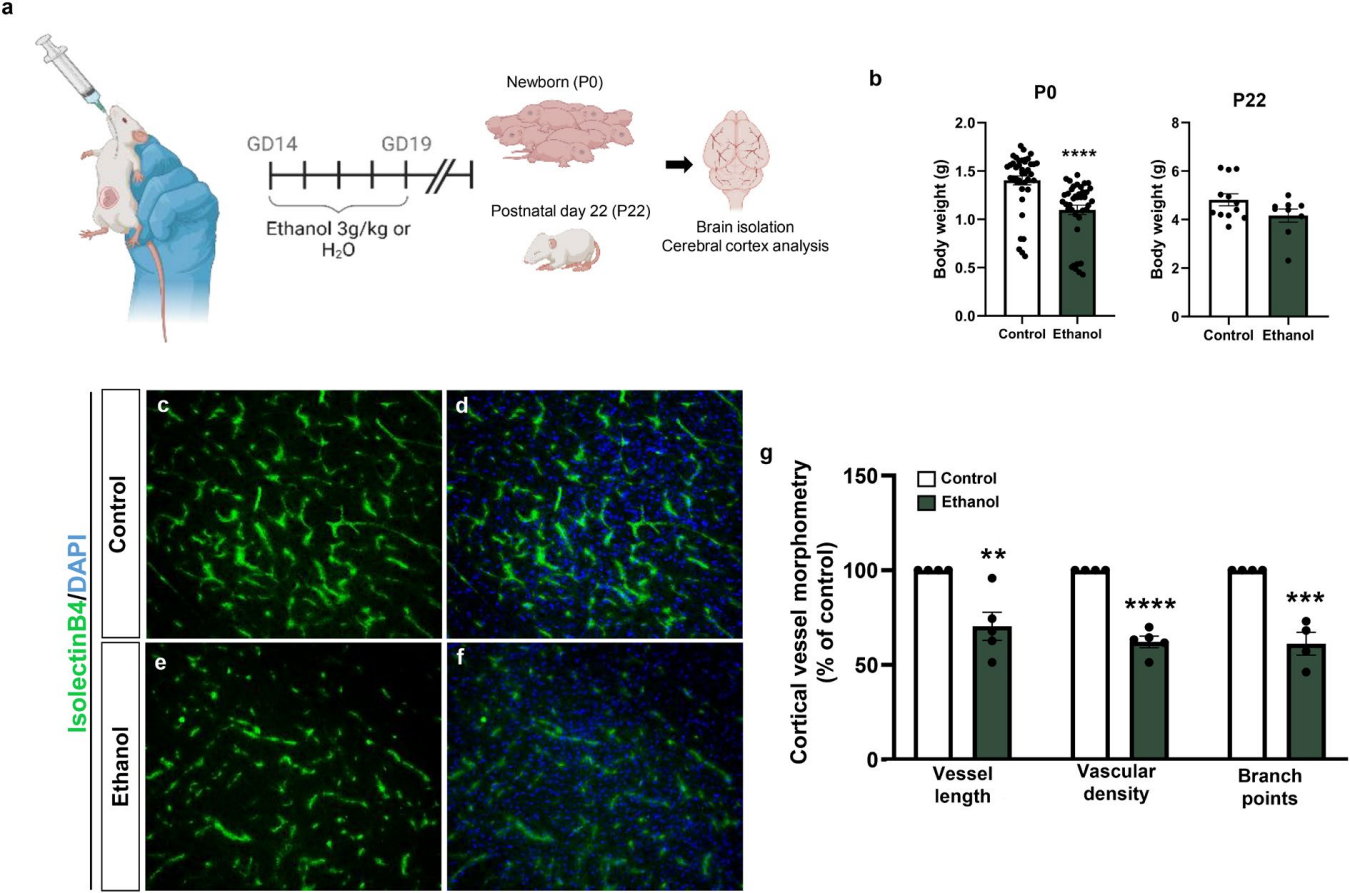
Long‐term PAE‐induced blood vessels morphological impairments in cerebral cortex. Swiss mice exposed to ethanol from gestational day 14 (GD14) to GD19 were weighed at birth (P0) and at postnatal day 22 (P22), after which brains were collected and processed for angiogenesis analysis by immunohistochemistry (a). The reduction in body weight observed at P0 confirms the fetal alcohol spectrum disorder (FASD) phenotype previously described by us. At P22, no significant differences in body weight were no longer detected between PAE and control mice (b). Immunofluorescence analyses revealed that ethanol‐exposed mice exhibited impaired cortical vascular development, characterized by reduced vessel length, density, and branching, as determined by Isolectin B4–FITC labeling (c–g). *p < 0.05, **p < 0.01, ***p < 0.001. Data are presented as mean ± SEM. *n* = 4 mice from 3 independent litters. P22, postnatal day 22; PAE, prenatal alcohol exposure.

Although PAE postnatal day 22 (P22) mice exhibited a trend toward reduced body weight, no statistically significant differences were detected compared with control animals, in contrast to newborn (P0) mice, which displayed a significant reduction in body weight (Figure 7a,b).

In contrast to the hypervascularized cortical phenotype previously observed in PAE newborn mice (P0) (Siqueira et al. 2021), the cerebral cortex of P22 mice exposed to ethanol exhibited an opposite phenotype, characterized by reduced vascular arborization, as revealed by Isolectin B4 labeling (Figure 7c–f). Morphometric analysis confirmed this hypovascularized phenotype, showing significant reductions of approximately 30% in vessel length, 38% in vascular density, and 39% in the number of branch points compared with controls (Figure 7c–g).

### 3.8 S‐Adhenosylmethionine Treatment Rescues Ethanol‐Induced Hypomethylation in Endothelial Cells

We have demonstrated that ethanol‐induced vascular morphological alterations persist into later stages of development. Moreover, our data indicate that endothelial exposure to ethanol promotes long‐lasting DNA hypomethylation, which is accompanied by altered DNMT activity and changes in DNMT protein labeling intensity levels. To further investigate whether the negative modulation of DNA methylation induced by ethanol is directly linked to DNMT function, we treated HBMEC cultures with ethanol in the presence or absence of the DNMT substrate S‐adenosylmethionine (SAM) for 24 h and quantified global 5‐methylcytosine (5mC) fluorescence intensity.

**FIGURE 8:**
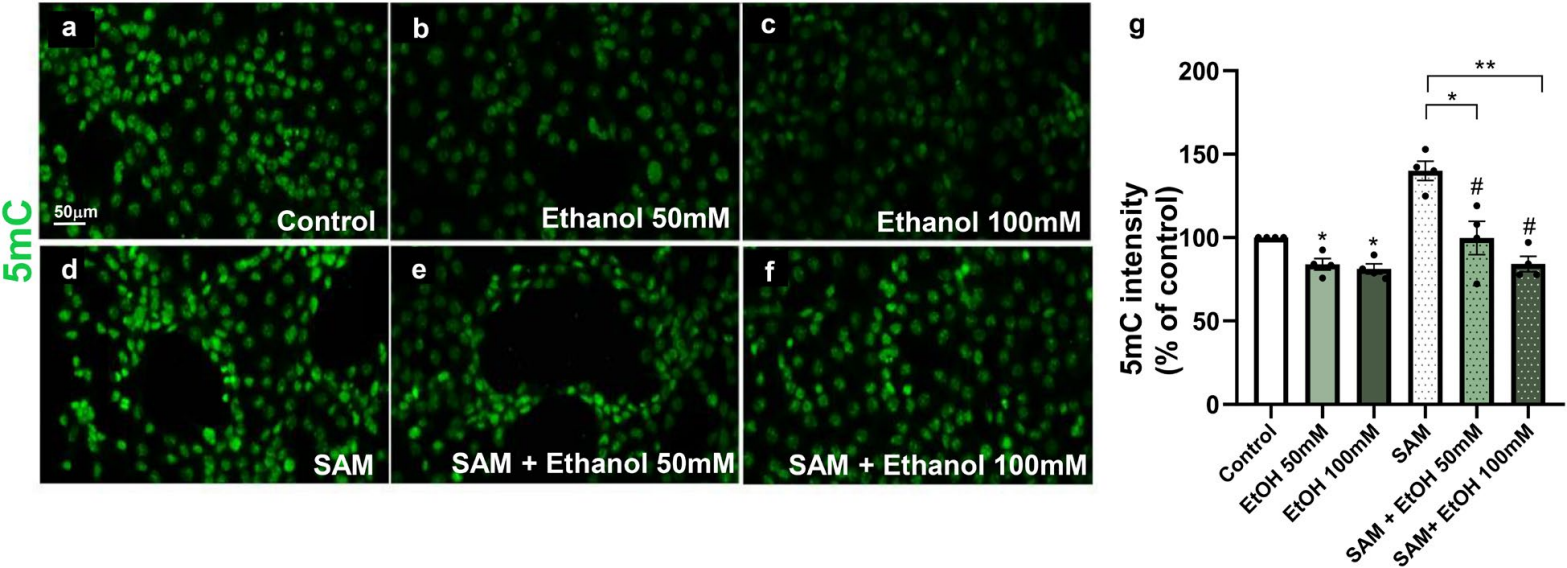
SAM prevents ethanol effects on DNA methylation in human brain endothelial cells. HBMEC cultures were treated with ethanol (50 or 100 mM) in the presence or absence of S‐adenosylmethionine (SAM; 50 μM) for 24 h and analyzed by immunocytochemistry for 5‐methylcytosine (5mC; green). Exposure to both ethanol concentrations significantly reduced total 5mC fluorescence intensity (a–c, g). Treatment with SAM alone significantly increased 5mC labeling intensity compared with controls (a, d, g). Treatment with SAM in the presence of both ethanol concentrations rescued 5mC levels to values comparable to those of control cells (a, e, f, g). *p < 0.05, **p < 0.005. Data are presented as mean ± SEM. *n* = 4 independent cell culture preparations. 5mC, 5‐methylcytosine; HBMEC, human brain microcapillary endothelial cells.

As previously observed, exposure to 50 and 100 mM ethanol significantly reduced 5mC fluorescence intensity (Figure 8a–c,g). Treatment with 50 μM SAM alone resulted in a significant 30% increase in global DNA methylation levels compared with controls (Figure 8d,g). Notably, addition of SAM in the presence of ethanol promoted the recovery of 5mC fluorescence intensity to levels comparable to those of control cells (Figure 8e–g).

## 4 Discussion

Previously, we demonstrated that PAE induces profound structural alterations in developing cortical blood vessels, characterized by excessive angiogenesis and changes in ZO‐1 and GLUT1 protein levels both in vivo and in vitro (Siqueira et al. 2021). Here, we show that these structural abnormalities are accompanied by functional deficits, including increased vascular permeability to 10‐kDa FITC–Dextran, as well as the accumulation and extravasation of plasma‐derived IgG from cerebral cortical blood vessels.

**FIGURE 9:**
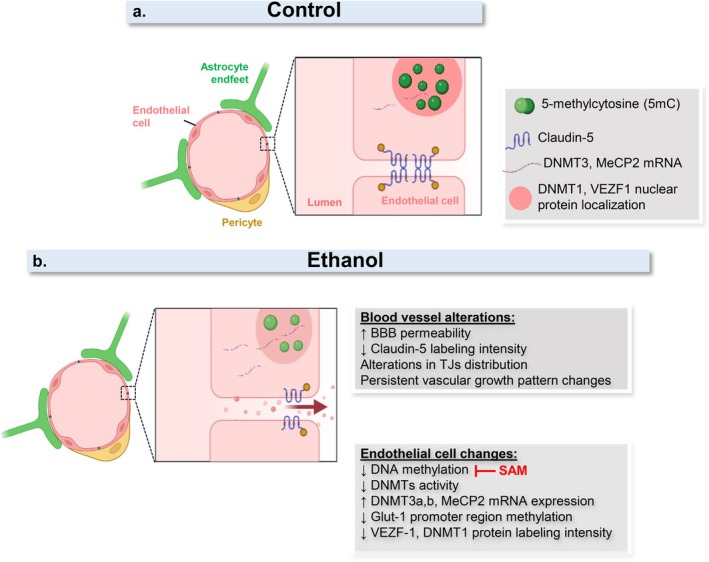
Impacts of ethanol exposure on brain endothelial cells and in a mouse model of PAE. (a) Schematic representation of blood–brain barrier (BBB) endothelial cells expressing basal levels of tight junction proteins, such as Claudin‐5, localized at the plasma membranes of neighboring cells. In the nuclei of human brain microcapillary endothelial cells (HBMECs) and mouse brain endothelial cells (MBECs) isolated from the prenatal alcohol exposure (PAE) mouse model, methylated DNA (5mC), DNA methyltransferases (DNMTs), methyl‐CpG binding protein 2 (MeCP2), and vascular endothelial zinc finger 1 (VEZF1) are depicted. (b) In the context of PAE, ethanol increases cortical vascular permeability, which is accompanied by reduced levels and altered spatial distribution of Claudin‐5 in newborn mice (P0). At later developmental stages (P22), vascular alterations persist, with reduced vascular arborization compared with control animals. In vitro, ethanol induces a global reduction in DNA methylation levels associated with decreased DNMT1 activity in HBMECs. In parallel, ethanol modulates the expression of DNMT3a/3b, MeCP2, and VEZF1, as well as the methylation status of promoter regions of glucose transporter 1 (*Glut‐1*) and *Claudin‐5* genes in a dose‐ and time‐dependent manner. Treatment with S‐adenosylmethionine (SAM) prevents ethanol‐induced DNA methylation changes in HBMECs. *Created with BioRender*.

By investigating the mechanisms underlying ethanol‐induced endothelial cell dysfunction, we demonstrated that ethanol promotes global DNA hypomethylation, which was successfully restored by SAM supplementation in vitro. DNA hypomethylated phenotype of endothelial DNA, was persistent even after withdrawal of ethanol treatment. These events were accompanied by reduced DNA methyltransferase activity and by alterations in the expression of DNMT isoforms and the transcriptional factors MeCP2 and VEZF1. Moreover, our results suggest that ethanol‐mediated modulation of the epigenetic machinery selectively affects the methylation status of promoter regions of genes essential for BBB function, which may contribute to the long‐lasting morphological and functional impairments observed in cerebral blood vessels later in development (Figure 9).

### 4.1 PAE Induces BBB Break in Cerebral Cortex

The proper growth and patterning of cerebral blood vessels during early embryonic development are essential for CNS formation and function. This importance extends beyond the vascular role in oxygen and nutrient delivery, as brain endothelial cells have been shown to actively regulate neural and glial development through the secretion of trophic factors (Da Silva et al. 2019). Blood vessels also provide instructive cues for the migration and differentiation of diverse cell populations during neurodevelopment (Paredes et al., 2018; Peguera et al., 2021). Consequently, disturbances in cerebral vascular growth, whether excessive or insufficient, have the potential to profoundly disrupt normal CNS development (Paredes et al., 2018; Siqueira and Stipursky 2022). In this context, PAE has been shown to alter vascular development and the positioning of oligodendrocyte progenitor cells in the developing brain (Brosolo et al. 2022). Moreover, experimental inhibition of angiogenesis during the first postnatal week in mice impairs neuroblast migration from the subventricular zone to the olfactory bulb (Angelidis et al., 2018), further highlighting the instructive role of the vasculature in brain maturation.

Excessive angiogenesis and increased vascular permeability have been reported in several pathological conditions (Carmeliet, 2003; Rigau et al., 2007), and we previously demonstrated alterations in the distribution of TJ proteins along cortical blood vessels in the developing CNS following PAE (Siqueira et al. 2021). Importantly, perturbations in BBB permeability have been shown to precede overt clinical manifestations in multiple neuropathological conditions, including Alzheimer's disease (Zaragoza 2020), and have been proposed as early biomarkers of cognitive dysfunction in humans (Nation et al. 2019).

Here, we confirm that an altered spatial distribution of Claudin‐5 is associated with increased vascular permeability to both plasma‐derived IgG and low‐molecular‐weight tracers such as FITC–Dextran. In control animals, serum immunoglobulins were confined to the lumen of Isolectin B4–labeled vessels, whereas in ethanol‐exposed animals we detected focal sites of IgG extravasation into the brain parenchyma. In addition to parenchymal leakage, suggesting an impairment in the integrity of the BBB induced by ethanol, we also observed a marked accumulation of IgG along the luminal walls of cortical capillaries. Similar IgG deposits have been described in animal models of diabetes, which also display increased angiogenesis, disrupted gliovascular interactions, loss of pericyte coverage, and increased astrocytic and microglial reactivity (Liu et al., 2021). Although gross BBB disruption was not detected in those models, it has been suggested that abnormal intravascular IgG deposition may impair microvascular perfusion and endothelial viability.

Consistent with this interpretation, our data suggest that early ethanol exposure induces both morphological and functional alterations in vascular growth patterns, resulting in enhanced IgG extravasation and abnormal luminal deposition, which may compromise cerebral perfusion. This cerebrovascular leakage was further corroborated by the intracardiac injection of a low‐molecular‐weight fluorescent tracer (10‐kDa FITC–Dextran), which revealed extensive diffusion into the brain parenchyma of ethanol‐exposed mice. Notably, tracer extravasation appeared to be more pronounced in capillaries exhibiting aberrant clusters of Claudin‐5.

Claudin‐5 is the most abundantly expressed TJ protein in brain endothelial cells, and its dysregulation has been directly linked to BBB breakdown. Its spatial organization is stabilized through interactions with cytoplasmic scaffolding proteins such as ZO‐1, which anchor Claudin‐5 to the actin cytoskeleton (Morita et al., 1999; Greene et al., 2019). We previously reported a similarly disrupted ZO‐1 distribution pattern in the cortical vasculature of animals exposed to alcohol in utero (Siqueira et al. 2021), suggesting a broader structural disorganization of TJ complexes between adjacent endothelial cells. Together, these findings support the notion that increased vascular permeability is closely associated with changes in both the levels and spatial distribution of TJ proteins.

In agreement with this hypothesis, we observed a significant reduction in *Claudin‐5* gene expression in the cerebral cortex of ethanol‐exposed mice. This suggests that, although Claudin‐5 appears agglomerated along the vessel walls, its overall abundance may be reduced and its structural integrity compromised by ethanol exposure. Such changes may collectively contribute to the impaired barrier function observed in the developing brain following PAE.

### 4.2 Ethanol Modifies the DNA Methylation Pattern of Brain Endothelial Cells

Substantial evidence indicates that epigenetic modifications play a critical role in ethanol‐induced alterations in glial and neuronal physiology (Ungerer et al. 2013; Basavarajappa and Subbanna 2016; Mandal et al. 2017; Siqueira and Stipursky 2022). In addition, our group previously demonstrated that direct exposure of control mouse brain endothelial cells (MBEC) to ethanol induces global changes in the levels of angiogenesis‐related molecules, along with pronounced impairments in BBB‐associated structural and functional properties (Siqueira et al. 2021). These observations raised the question of whether ethanol exerts its effects on endothelial cells by modulating global gene expression programs.

DNA methylation is a central epigenetic mechanism regulating endothelial gene expression and is involved in processes such as proliferation, differentiation, and angiogenesis across multiple contexts (Goyal and Goyal 2019; Zhang et al. 2016). Here, we show that exposure to different concentrations of ethanol induces global DNA hypomethylation in endothelial cells, leading to reductions in the number, volume, and fluorescence intensity of 5mC‐positive chromocenters in HBMECs. Notably, we also demonstrate that ethanol induces persistent changes in the DNA methylation of these cells, as reduced methylation levels were still observed 24 h after ethanol withdrawal. This global hypomethylation may result in increased endothelial gene expression and could represent a mechanistic basis for the elevated production of angiogenic molecules observed in response to ethanol exposure.

Importantly, we further show that MBECs isolated from the cerebral cortex of newborn animals from the FASD model exhibited a markedly greater reduction in 5mC fluorescence intensity than HBMECs acutely exposed to ethanol in vitro. These findings suggest that prenatal ethanol exposure may trigger long‐lasting neurovascular dysfunction through epigenetic dysregulation. Moreover, the hypomethylated state persisted after tissue dissociation and several days of in vitro culture, indicating that these epigenetic changes are stable and long‐lasting.

Maintenance of DNA methylation patterns relies primarily on DNMT1 activity, which ensures the transmission of methylation marks during DNA replication. Although we did not observe significant changes in *DNMT1* mRNA levels, we detected a transient reduction in DNMT1 protein fluorescence intensity within the first 2 h of ethanol exposure, which recovered after 24 h. In parallel, ethanol exposure significantly reduced overall DNMT enzymatic activity in endothelial cells, an effect that persisted after 24 h. Although the enzymatic assay employed here did not distinguish among individual DNMT isoforms, these data suggest that the observed loss of cytosine methylation may result, at least in part, from impaired DNMT activity.

Alternatively, DNA hypomethylation could also be driven by active demethylation mechanisms mediated by ten‐eleven translocation (TET) proteins, which catalyze the conversion of 5‐methylcytosine to 5‐hydroxymethylcytosine (5hmC) and subsequent demethylation through the base excision repair pathway (Tsiouplis et al. 2021). Previous studies have shown that prenatal ethanol exposure reduces TET expression and 5hmC levels in the dentate gyrus of the hippocampus, thereby impairing neuronal maturation (Chen et al. 2013). Thus, it will be important in future studies to determine whether ethanol similarly modulates TET expression and activity in endothelial cells, potentially contributing to the hypomethylation observed here.

Interestingly, we observed a significant upregulation of *DNMT3a* expression following ethanol exposure across multiple concentrations and time points. DNMT3a has been implicated in the regulation of endothelial function and vessel formation in various experimental models (Gao et al. 2011; Zhang et al. 2016). Increased DNMT3a expression has been shown to induce hypermethylation of the *Klf4* promoter, resulting in reduced *Klf4* transcription (Jiang et al. 2014). KLF4 is a transcription factor that regulates the expression of multiple genes critical for angiogenesis (Hale et al. 2014). In retinal endothelial cells, KLF4 directly binds to the *VEGF* promoter, promoting its transcription and thereby enhancing cell proliferation, migration, and tubulogenesis (Wang et al. 2015). Thus, DNMT3a‐mediated suppression of *Klf4* expression could have important consequences for vascular growth and remodeling.

Although we observed an overall reduction in global DNMT activity, the differential regulation of DNMT3a in HBMECs suggests that this isoform may selectively influence the transcription of specific angiogenesis‐related genes. This complex interplay between global hypomethylation and locus‐specific hypermethylation may contribute to the aberrant vascular phenotypes induced by ethanol.

Recent evidence indicates that endothelial cells overexpressing DNMT3a and DNMT3b release exosomes with proangiogenic activity and an altered proteomic profile enriched in molecules associated with vascular damage (Vasishta et al. 2024). DNMT3a and DNMT3b are known to establish *de novo* DNA methylation patterns by transferring methyl groups to previously unmethylated cytosines (Saravanaraman et al. 2020). Thus, the upregulation of these DNMT isoforms observed following ethanol exposure may ultimately reshape the endothelial transcriptomic profile, contributing to the hypervascularized phenotype and the endothelial dysfunctions described here.

In addition to the essential roles of DNMTs in the maintenance and establishment of DNA methylation patterns, we also evaluated the effects of ethanol on the transcriptional regulators MeCP2 and VEZF1. MeCP2 binds to methylated CpG sites and mediates transcriptional repression by recruiting corepressor complexes to DNA (Zachariah and Rastegar 2012; Vuu et al. 2023). This protein regulates the expression of numerous genes and influences multiple developmental processes, including neurogenesis, neuronal maturation, and synaptogenesis (Jin et al. 2017; Gulmez Karaca et al. 2019; Albizzati et al. 2024). Notably, in a *Mecp2*‐null mice model of Rett syndrome, increased BBB permeability has been associated with dysregulated expression of genes encoding key BBB structural and functional components, including Occludin, Claudins 3/5/12, Mpdz, Jam2, and Aquaporin4 (Pepe et al. 2023).

Here, we found that acute exposure to ethanol for 2 h reduced MeCP2 expression without altering the global DNA methylation profile. In contrast, prolonged exposure significantly increased MeCP2 expression, indicating that the endothelial response to ethanol is both dose‐ and time‐dependent. Similar increases in MeCP2 expression have been reported in neural stem cell cultures exposed to ethanol for extended periods, where they were associated with reductions in 5mC levels (Liyanage et al. 2015). These findings suggest a complex feedback loop between DNA methylation dynamics and MeCP2‐mediated transcriptional regulation.

We also examined the role of the Krüppel‐like zinc finger transcription factor VEZF1, a key regulator of angiogenesis and vascular development. VEZF1 has been shown to modulate *Dnmt3b* expression in embryonic stem cells, indirectly shaping selective DNA methylation patterns and thereby influencing genome‐wide gene expression (Gowher et al. 2008). Furthermore, VEZF1 acts as an insulator protein that regulates the anti‐angiogenic factor *Cited2* by blocking inappropriate and nonspecific interactions of promoters with *cis*‐located enhancers, preventing aberrant promoter activation (AlAbdi et al. 2018). Recent studies have demonstrated that prenatal ethanol exposure suppresses VEZF1 expression through the upregulation of miR‐150‐5p, leading to impaired endothelial migration and tube formation (Perales et al., 2022). These findings reinforce the idea that VEZF1 dysregulation contributes to cortical microvascular abnormalities and may directly underlie some of the neurovascular deficits observed in FASD.

Collectively, our data indicate that ethanol induces DNA methylation changes in brain endothelial cells, likely through the deregulation of key components of the epigenetic machinery, including DNMTs, MeCP2, and VEZF1. We further demonstrate that these alterations are associated with a marked increase in methylation levels at the *Claudin‐5* promoter following 24 h of exposure to 100 mM ethanol. Importantly, increased methylation of the *Claudin‐5* promoter has been described as a hallmark of BBB dysfunction in Alzheimer's disease (Hüls et al. 2022) and stroke (Phillips et al. 2023). Although DNMT1 protein levels transiently decreased after 2 h of ethanol exposure, *Dnmt1* mRNA expression remained unchanged at both 2 and 24 h, suggesting that *Dnmt1*, despite being highly expressed in HBMECs, may not be essential to *Claudin‐5* promoter methylation. In contrast, however, *Dnmt3a and 3b* expression was increased by 100 mM ethanol treatment. Given that *Dnmt3* isoforms primarily establish new methylation patterns during development and disease (Saravanaraman et al. 2020), it is plausible that, at higher ethanol concentrations, the *Claudin‐5* promoter becomes a specific target of pathological methylation. This could contribute to the altered organization and distribution of TJ proteins in cortical blood vessels, ultimately leading to impaired vascular permeability.

We also observed that MeCP2 and VEZF1 expression increased in response to 50 mM ethanol, although methylation levels at the *Claudin‐5* promoter remained unchanged in this condition. While direct evidence linking these transcription factors to *Claudin‐5* regulation in the CNS is limited, MeCP2 binding to the *Claudin‐6* promoter has been shown to increase DNA methylation and promote a migratory and invasive phenotype in MCF‐7 breast cancer cells (Liu et al. 2016). Thus, we cannot exclude the possibility that MeCP2 and VEZF1 contribute to endothelial promoter methylation and BBB destabilization at lower ethanol concentrations.

Although *Claudin‐5* promoter methylation appears to be dose‐dependent, both 50 and 100 mM ethanol robustly reduced methylation levels at the *GLUT1* promoter. This suggests that ethanol‐induced epigenetic changes may compromise glucose transport in endothelial cells. Glucose is the primary energy substrate for the CNS and is essential for maintaining cerebral metabolic homeostasis. We previously demonstrated that short‐term ethanol exposure inhibits glucose uptake in HBMECs (Siqueira et al. 2021), indicating that even brief insults may impair energy supply to the brain. Supporting this, GLUT1 deficiency in mice has been linked to synaptic dysregulation, mitochondrial morphological abnormalities, and compensatory changes in GLUT1 and GLUT3 expression in Alzheimer's disease models (Pang et al. 2019).

As mentioned, we observed that MeCP2 and VEZF1 levels increased following treatment with 50 mM ethanol but remained unchanged at higher concentrations. This contrasts with previous findings showing that both acute ethanol exposure and chronic PAE reduce VEZF1 expression in brain endothelial cells, leading to angiogenic deficits (Perales et al., 2022). Given the proposed role of VEZF1 as an epigenetic insulator and regulator of DNA methylation (Gowher et al. 2008; AlAbdi et al. 2018), and the emerging evidence implicating MeCP2 in BBB regulation, it is likely that these transcription factors are critical mediators of ethanol‐induced epigenetic remodeling. Together, our findings reinforce the concept that ethanol‐induced dysregulation of *Glut1* expression and epigenetic control in endothelial cells may exert detrimental effects on energy supply to CNS cells.

### 4.3 Ethanol‐Induced Vascular Impairments Are Persistent

A growing body of evidence indicates that epigenetic reprogramming can induce long‐term changes in cellular function and exert transgenerational effects in the CNS (Bale 2015; Breton et al. 2021). In the present study, we show that ethanol induces persistent alterations in endothelial DNA methylation, as reduced methylation levels were still evident after ethanol withdrawal in HBMECs and after several days of in vitro culture in MBECs derived from PAE animals. Notably, the magnitude of 5mC reduction observed 24 h after ethanol withdrawal in HBMEC cultures was even greater than that detected during the exposure period itself. Furthermore, isolation of cerebral cortex microvascular fragments followed by several days of culture yielded MBECs that exhibited a more pronounced reduction in 5mC fluorescence intensity than that observed in HBMECs acutely exposed to ethanol. These findings suggest that ethanol initiates epigenetic alterations that continue to propagate over time, resulting in a stable and progressive loss of DNA methylation. Such stability is consistent with the heritable nature of epigenetic marks and supports the idea that prenatal ethanol exposure can induce long‐lasting reprogramming of endothelial cells.

In parallel with these persistent epigenetic alterations, we also observed long‐term changes in vascular growth patterns in mice at postnatal day 22 (P22). In murine models, angiogenic activity markedly decreases after birth, and capillary branching reaches a plateau between postnatal days 15 and 25, coinciding with the acquisition of a quiescent endothelial phenotype and vascular maturation (Harb et al. 2013). Despite this developmental stabilization, PAE P22 mice displayed significant deficits in cortical vascular network organization. These changes in the vascular growth pattern throughout development may reflect altered vascular remodeling, characterized either by excessive pruning of newly formed vessels or by impaired expression of vascular maturation factors.

During postnatal development, angiogenesis initially generates a surplus of vessels, which are subsequently refined through pruning to establish a mature and functional vascular network (Korn and Augustin 2015). Vessel regression is driven by multiple mechanisms, including endothelial apoptosis due to altered expression of survival factors such as VEGF, as well as vascular obstruction and reduced perfusion (Zhang et al. 2018). Importantly, nonperfused vessels are particularly susceptible to regression. As demonstrated here, the cerebral cortex vasculature of newborn mice exposed to ethanol exhibited pronounced defects in vascular integrity and likely impaired perfusion. Thus, these observations support a model in which ethanol initially promotes excessive formation of structurally and functionally defective vessels during early development. Upon removal of the insult, these dysfunctional vessels may undergo accelerated regression and elimination, ultimately leading to the hypovascularized phenotype observed later in development.

Interestingly, Bake and colleagues followed PAE and control offspring into adulthood and observed opposite effects on cerebral blood flow responses to transient ischemic challenges. Young adult PAE offspring (3 months of age) exhibited significantly increased carotid blood flow, indicative of an altered vasoconstrictive response. In contrast, in mature adults (12 months of age), carotid blood flow was reduced compared with control offspring, suggesting that PAE may ultimately lead to cranial vascular hypoperfusion later in life (Bake et al. 2017). Complementing these findings, a recent and robust human study showed that children aged 3–8 years who were prenatally exposed to alcohol and subjected to MRI analysis exhibited reduced cerebral blood flow compared with unexposed controls, with the most pronounced effects observed in subcortical and medial frontal regions (Ghasoub et al. 2025). Furthermore, a large longitudinal study evaluating ophthalmological outcomes in eastern European and Swedish children with FASD, followed from childhood into adulthood, revealed that early‐detected abnormalities, including astigmatism, impaired stereoacuity, heterotropia, optic nerve hypoplasia, and increased tortuosity of retinal vessels, persisted into adulthood (Gyllencreutz et al. 2020). Together, these data strongly suggest that early‐life vascular abnormalities induced by PAE may persist and evolve into long‐term cerebrovascular dysfunction.

Alterations in CNS vascular development have increasingly been associated with late‐onset dysfunctions, some of which increase the risk of cerebrovascular disease (Bake et al. 2022; Lunde et al. 2016). Notably, recent work has provided the first direct evidence that PAE significantly worsens stroke outcomes in middle‐aged rats. In adulthood, Bake and colleagues demonstrated a greater dysregulation of inflammatory and vascular responses in PAE animals subjected to middle cerebral artery occlusion (MCAo). These animals exhibited elevated levels of pro‐inflammatory cytokines, including IL‐6, IL‐17, and RANTES, in the ischemic hemisphere. In addition, immune cell profiles were altered, with a reduced CD4^+^/CD8^+^ ratio in circulation, indicating a weakened immune response and increased vulnerability to secondary complications (Bake et al. 2025). Collectively, these findings suggest that PAE not only induces acute neurovascular alterations but also increases the risk of cerebrovascular dysfunction later in life. However, it remains unknown whether the mechanisms underlying these long‐term vascular consequences involve epigenetic modifications accumulated during early development, and whether such changes could be therapeutically targeted. Our data indicate that PAE induces profound alterations in cortical angiogenesis and that vascular abnormalities persist into later developmental stages, as evidenced by the reduced arborization observed at P22. Whether DNA methylation changes in vascular endothelial cells persist into adulthood and whether these changes contribute to long‐term neurovascular dysfunction or increased susceptibility to cerebrovascular disease remains an important question for future studies.

In vitro, we observed that treatment with S‐adenosylmethionine (SAM) rescued ethanol‐induced DNA hypomethylation in endothelial cells, restoring methylation levels to those observed in control conditions. One‐carbon metabolism, or methyl group transfer, is a fundamental cellular process involved in the synthesis and methylation of numerous substrates and in the regulation of gene expression. SAM is a central intermediate of the activated methionine cycle and serves as the universal methyl donor for most methyltransferase reactions (Kalhan 2013). In humans, three catalytically active DNA methyltransferases, DNMT1, DNMT3A, and DNMT3B, use SAM to transfer a methyl group to the carbon‐5 position of cytosine residues.

Disruptions in the methylation cycle can arise from genetic alterations, exposure to toxic chemicals or nutritional deficiencies, and have been implicated in the pathogenesis of diverse disorders, including cancer, diabetes mellitus, atherosclerosis, cardiovascular disease, congenital malformations, and neurodegenerative disorders (Davletgildeeva and Kuznetsov 2024). Accordingly, epigenetic therapies have recently emerged as promising strategies to restore cellular functions disrupted by aberrant epigenetic regulation. For example, in a rat model of cerebral ischemia/reperfusion injury, treatment with the DNMT inhibitor 5‐aza‐2′‐deoxycytidine improved neurological outcomes as well as cognitive, social, and spatial memory performance (Shi et al. 2023).

S‐adenosylmethionine (SAM) has been investigated as a potential therapeutic and adjuvant molecule in a wide range of disorders, including rheumatic diseases, Alzheimer's disease, depression, and liver pathologies, among others (Hardy et al., 2003; Sun et al. 2024). Anier and colleagues reported that SAM pretreatment modifies cocaine‐induced gene expression and is associated with reduced *DNMT3a* and *DNMT3b* expression in the nucleus accumbens (Anier et al. 2013). In a rat model of chronic cerebral hypoperfusion combined with intrahippocampal Aβ injection, SAM administration rescued brain‐derived neurotrophic factor (BDNF) mRNA and protein levels by positively modulating the expression of BDNF exons IV and VI. These findings suggest that SAM exerts a neuroprotective role by enhancing endogenous BDNF expression and may represent a promising therapeutic candidate for Alzheimer's disease (Li et al. 2016).

Because SAM levels are key regulators of multiple biochemical pathways, modulation of its availability has also been proposed as a novel therapeutic strategy for several diseases (Davletgildeeva and Kuznetsov 2024). Methyl donor supplementation, including SAM, has been shown to ameliorate the teratogenic effects of alcohol, reversing DNA methylation changes associated with prenatal alcohol exposure in animal models and improving growth, neurodevelopmental, and behavioral outcomes in both animals and humans (Gutherz et al. 2022). Thus, epigenetic drugs that target the DNA methylation machinery may represent promising tools for rescuing neurovascular dysfunctions induced by fetal ethanol exposure and for preventing the increased risk of vascular disease later in life.

Taken together, our findings highlight the profound effects of PAE on epigenetic regulation in brain endothelial cells (Figure 9). Our data point to ethanol‐induced alterations in DNA methylation patterns as a key mechanism underlying BBB dysfunction. Changes in DNMT isoform expression and activity, together with the modulation of additional epigenetic regulators such as MeCP2 and VEZF1, may ultimately alter endothelial promoter methylation profiles, reinforcing the deleterious consequences of alcohol consumption during pregnancy and contributing to persistent neurovascular impairments.

Because BBB formation is a critical step in CNS maturation and function, early‐life exposure to teratogenic substances such as ethanol may disrupt vascular–neural communication and compromise long‐term brain homeostasis. Importantly, our data show that SAM treatment prevents ethanol‐induced global hypomethylation in endothelial cells, suggesting that one‐carbon metabolism plays a central role in the regulation of vascular function. These findings identify SAM as a potential pharmacological target for future investigations aimed at mitigating ethanol‐induced pathological changes in endothelial cells.

In conclusion, this study advances our understanding of epigenetic reprogramming as a mechanistic link between prenatal alcohol exposure and persistent neurovascular dysfunction. Moreover, it supports the concept that targeting epigenetic pathways may represent a promising therapeutic strategy to counteract the long‐term neurological and structural impairments observed in individuals with FASD.

## Author Contributions

**Michele Siqueira:** investigation, methodology, visualization, formal analysis, data curation. **Matheus Barros:** investigation, methodology, visualization, formal analysis, data curation. **Paula Lacerda Almeida:** investigation, visualization, data curation, formal analysis, methodology. **Luiza dos Santos Heringer:** methodology. **Henrique Rocha Mendonça:** writing – review and editing, supervision, resources. **Flávia Carvalho Alcantara Gomes:** writing – review and editing, supervision. **Joice Stipursky:** conceptualization, investigation, funding acquisition, writing – original draft, methodology, project administration, resources, supervision, data curation.

## Funding

This work was supported by Fundação Carlos Chagas Filho de Amparo à Pesquisa do Estado do Rio de Janeiro, E‐26/201.427/2021, E‐26/210.326/2019.

## Conflicts of Interest

The authors declare no conflicts of interest.

## Supporting information


**Table S1:** Compilation of all statistical data.

## Data Availability

The datasets generated during the current study are available from the corresponding author on reasonable request.
