# Supplementary material for: Ethanol Alters DNMT1/3a/3b Expression Profile, Promotes Persistent DNA Hypomethylation in Human Brain Endothelial Cells and Impairs Late Cortical Angiogenesis
Source: J Neurochem. 2026 Feb 10;170(2):e70375. doi: 10.1111/jnc.70375 (PMC12891768; doi:10.1111/jnc.70375)
Supplement: Supplementary file 1 — Table S1: Compilation of all statistical data. [file JNC-170-0-s001.pdf]

Ethanol alters DNMT1/3a/3b expression profile, promotes persistent DNA hypomethylation in human brain endothelial cells and impairs late cortical angiogenesis.

Authors: Michele Siqueira[2], Matheus Barros[1], Paula Lacerda Almeida[1], Luiza dos Santos Heringer[3], Henrique Rocha Mendonça[3], Flávia Carvalho Alcantara Gomes[2], Joice Stipursky[1].

[1]Laboratório de Biologia das Interações Neurovasculares, Instituto de Ciências Biomédicas, Universidade Federal do Rio de Janeiro

[2]Laboratório de Neurobiologia Celular, Instituto de Ciências Biomédicas, Universidade Federal do Rio de Janeiro

[3]Laboratório de Neurodegeneração e Reparo, Faculdade de Medicina, Universidade Federal do Rio de Janeiro

Corresponding author: Joice Stipursky

joice@icb.ufrj.br, jstipursky@gmail.com

Supplementary table 1.

FIGURE 1		test	Mean ± SEM	t	df	P value	one- or two-tailed
**Figure 1i**	Claudin-5 mRNA	Unpaired t test	Control (1.000 ± 0.000); Ethanol (0.5523 ± 0.1566)	3.168	9	0.0114	Two-tailed

FIGURE 2		test	Mean ± SEM	P value	F value	df between	df within
**Figure 2h**	5mC intensity 2h	One-way anova	Control (100.0 ± 0.0); 50mM (89.11 ± 22.99); 100mM (95.97 ± 27.61)	0.9328	0.07039	2	6
**Figure 2h**	5mC intensity 24h	One-way anova	Control (100.0 ± 0.0); 50mM (85.42 ± 4.13); 100mM (88.86 ± 2.76)	0.0058	9.016	2	10
**Figure 2k**	Number (chromocenters organization 2h)	One-way anova	Control (100.0 ± 0.0); 50mM (81.34 ± 11.48); 100mM (70.71 ± 14.25)	0.2201	1.969	2	6
**Figure 2k**	Volume (chromocenters organization 2h)	One-way anova	Control (100.0 ± 0.0); 50mM (73.70 ± 16.39); 100mM (67.21 ± 20.87)	0.3432	1.285	2	6
**Figure 2k**	Intensity (chromocenters organization 2h)	One-way anova	Control (100.0 ± 0.0); 50mM (79.45 ± 9.374); 100mM (65.18 ± 18.93)	0.2084	2.060	2	6
**Figure 2k**	Number (chromocenters organization 24h)	One-way anova	Control (100.0 ± 0.0); 50mM (78.91 ± 4.056); 100mM (62.57 ± 3.352)	0.0004	38.16	2	6
**Figure 2k**	Volume (chromocenters organization 24h)	One-way anova	Control (100.0 ± 0.0); 50mM (74.07 ± 4.632); 100mM (59.91 ± 1.719)	0.0002	50.79	2	6

**Figure 2k**	Intensity (chromocenters organization 24h)	One-way anova	Control (100.0 ± 0.0); 50mM (82.12 ± 3.298); 100mM (72.33 ± 1.364)	0.0002	46.36	2	6

**FIGURE 3**		**test**	**Mean ± SEM**	**P value**	**F value**	**df between**	**df within**
**Figure 3a**	Control DNMT mRNA	One-way anova	DNMT1 (100.0 ± 0.0); DNMT3a (0.07800 ± 0.02699); DNMT3b (0.2144 ± 0.1025)	<0.0001	66.17	2	12
**Figure 3b**	DNMT activity	One-way anova	Control (100.0 ± 0.0); 50mM (0.6775 ± 0.01382); 100mM (0.5271 ± 0.1511)	0.0038	12.06	2	8
**Figure 3i**	DNMT intensity 2h	One-way anova	Control (100.0 ± 0.0); 50mM (79.71 ± 9.785); 100mM (65.49 ± 6.423)	0.0306	6.586	2	6
**Figure 3i**	DNMT intensity 24h	One-way anova	Control (100.0 ± 0.0); 50mM (81.80 ± 13.32); 100mM (104.4 ± 22.32)	0.6044	0.5366	2	8
**Figure 3j**	DNMT1 2h	One-way anova	Control (100.0 ± 0.0); 50mM (0.7899 ± 0.02784); 100mM (1.301 ± 0.3478)	0.2492	1.628	2	9
**Figure 3j**	DNMT3a 2h	One-way anova	Control (100.0 ± 0.0); 50mM (0.4986 ± 0.1915); 100mM (2.714 ± 0.6473)	0.0161	8.888	2	6
**Figure 3j**	DNMT3b 2h	One-way anova	Control (100.0 ± 0.0); 50mM (1.099 ± 0.07794); 100mM (2.362 ± 0.5561)	0.013	7.843	2	8
**Figure 3j**	DNMT1 24h	One-way anova	Control (100.0 ± 0.0); 50mM (2.239 ± 0.7195); 100mM (1.601 ± 0.4130)	0.2646	1.673	2	6
**Figure 3j**	DNMT3a 24h	One-way anova	Control (100.0 ± 0.0); 50mM (2.132 ± 0.3349); 100mM (1.964 ± 0.02325)	0.0125	9.937	2	6
**Figure 3j**	DNMT3b 24h	One-way anova	Control (100.0 ± 0.0); 50mM (1.940 ± 0.4033); 100mM (2.814 ± 0.8172)	0.1021	2.972	2	9
**Figure 3k 2h**	MECP2 2h	One-way anova	Control (100.0 ± 0.0); 50mM (0.5163 ± 0.1243); 100mM (0.4394 ± 0.1039)	0.0044	10.55	2	9
**Figure 3k 24h**	MECP2 24h	One-way anova	Control (100.0 ± 0.0); 50mM (3.061 ±0.8071); 100mM (1.143 ± 0.1789)	0.024	5.812	2	9
**Figure 3o**	VEZF-1	One-way anova	Control (100.0 ± 0.0); 50mM (129.7 ± 14.43); 100mM (87.60 ± 10.53)	0.0239	5.179	2	12

**FIGURE 4**		**test**	**Mean ± SEM**	**P value**	**F value**	**df between**	**df within**
**Fig 4b**	Claudin-5 MeDIP-PCR	One-way anova	Control (100.0 ± 0.0); 50mM (0.7938 ± 0.2039); 100mM (2.342 ± 0.4054)	0.0047	10.29	2	9
**Fig 4b**	Glut-1 MeDIP-PCR	One-way anova	Control (100.0 ± 0.0); 50mM (0.1723 ± 0.08646); 100mM (0.3303 ± 0.1637)	0.0008	23.53	2	7

**FIGURE 5**		**test**	**Mean ± SEM**	**P value**	**F value**	**df between**	**df within**

Figure 5e	5mC intensity rescue	One-way anova	Control (100.0 ± 0.0); 50mM (65.94 ± 7.461); 100mM (73.48 ± 0.5080)	0.0008	17.16	2	9
Figure 5f	Number (chromocenters organization)	One-way anova	Control (100.0 ± 0.0); 50mM (57.87 ± 9.565); 100mM (54.47 ± 6.165)	0.0014	14.90	2	9
Figure 5f	Volume (chromocenters organization)	One-way anova	Control (100.0 ± 0.0); 50mM (51.75 ± 8.918); 100mM (53.49 ± 7.624)	0.001	16.32	2	9
Figure 5f	Intensity (chromocenters organization)	One-way anova	Control (100.0 ± 0.0); 50mM (60.92 ± 15.43); 100mM (62.31 ± 9.303)	0.0251	6.052	2	8

FIGURE 6		test	Mean ± SEM	t	df	P value	one- or two-tailed
**Fig 6l**	5mC intensity	Unpaired t test	Control (1.000 ± 0.000); Ethanol (42.61 ± 14.67)	3.912	4	0.0174	Two-tailed

FIGURE 7		test	Mean ± SEM	t	df	P value	one- or two-tailed
**Figure 7b**	Body weight P0	Unpaired t test	Control (1.404 ± 0.04620); Ethanol (1.098 ± 0.04934)	4.525	78	<0.0001	Two-tailed
**Figure 7b**	Body weight P22	Unpaired t test	Control (100.0 ± 0.0); Ethanol (86.00 ± 15.95)	1.769	19	0.0929	Two-tailed
**Figure 7g**	Vessel length	Unpaired t test	Control (100.0 ± 0.0); Ethanol (70.40 ± 7.397)	3.529	7	0.0096	Two-tailed
**Figure 7g**	Vascular density	Unpaired t test	Control (100.0 ± 0.0); Ethanol (62.15 ± 3.033)	11.01	7	<0.0001	Two-tailed
**Figure 7g**	Branch points	Unpaired t test	Control (100.0 ± 0.0); Ethanol (61.15 ± 6.008)	6.467	6	0.0006	Two-tailed

FIGURE 8		test	Mean ± SEM	P value	F value	df between	df within
**Figure 8g**	5mC intensity	One-way anova	Control (100.0 ± 0.0); 50mM (83.93 ± 3.42); 100mM (81.25 ± 3.03); SAM (140.01 ± 5.76)	0.0015	55.65	3	3

FIGURE 8		test	Mean ± SEM	P value	q	DF	
	5mC intensity	Two-way anova	Control (100.0 ± 0.0); Ethanol 50mM + SAM (99.74 ± 9.98)	0.9996	0.03613	6	
	5mC intensity	Two-way anova	Control (100.0 ± 0.0); Ethanol 100mM + SAM (84.18 ± 4.54)	0.3313	2.206	6	
	5mC intensity	Two-way anova	SAM (140.01 ± 5.76); Ethanol 50mM + SAM (99.74 ± 9.98)	0.0342	4.782	6	
	5mC intensity	Two-way anova	SAM (130.5.0 ± 0.0); Ethanol 100mM + SAM (84.18 ± 4.54)	0.0080	6.630	6	
